# A virulence-associated filamentous bacteriophage of *Neisseria meningitidis* increases host-cell colonisation

**DOI:** 10.1371/journal.ppat.1006495

**Published:** 2017-07-13

**Authors:** Emmanuelle Bille, Julie Meyer, Anne Jamet, Daniel Euphrasie, Jean-Philippe Barnier, Terry Brissac, Anna Larsen, Philippe Pelissier, Xavier Nassif

**Affiliations:** 1 Institut Necker-Enfants Malades, INSERM U1151, CNRS UMR 8253, Paris, France; 2 Université Paris Descartes, Paris, France; 3 Service de Microbiologie, Hôpital Necker-Enfants Malades, Assistance Publique-Hôpitaux de Paris, Paris, France; 4 Service de Chirurgie Reconstructrice et Plastique, Fondation Hôpital Saint Joseph, Paris, France; Emory University School of Medicine, UNITED STATES

## Abstract

*Neisseria meningitidis* is a commensal of human nasopharynx. In some circumstances, this bacteria can invade the bloodstream and, after crossing the blood brain barrier, the meninges. A filamentous phage, designated MDAΦ for Meningococcal Disease Associated, has been associated with invasive disease. In this work we show that the prophage is not associated with a higher virulence during the bloodstream phase of the disease. However, looking at the interaction of *N*. *meningitidis* with epithelial cells, a step essential for colonization of the nasopharynx, we demonstrate that the presence of the prophage, via the production of viruses, increases colonization of encapsulated meningococci onto monolayers of epithelial cells. The analysis of the biomass covering the epithelial cells revealed that meningococci are bound to the apical surface of host cells by few layers of heavily piliated bacteria, whereas, in the upper layers, bacteria are non-piliated but surrounded by phage particles which (i) form bundles of filaments, and/or (ii) are in some places associated with bacteria. The latter are likely to correspond to growing bacteriophages during their extrusion through the outer membrane. These data suggest that, as the biomass increases, the loss of piliation in the upper layers of the biomass does not allow type IV pilus bacterial aggregation, but is compensated by a large production of phage particles that promote bacterial aggregation via the formation of bundles of phage filaments linked to the bacterial cell walls. We propose that MDAΦ by increasing bacterial colonization in the mucosa at the site-of-entry, increase the occurrence of diseases.

## Author summary

Bacteriophages are bacterial viruses, which in some cases encode for virulence factors and increase bacterial virulence. Comparative genomic of several strains of *Neisseria meningitidis*, a major human pathogen, identified the presence of an 8kb prophage in strains belonging to invasive clonal complexes. The analysis of this filamentous bacteriophage, designated MDA for Meningococcal Disease Associated (MDAΦ) did not reveal any obvious virulence factors responsible for an increase invasiveness of strains carrying this prophage. Using our animal model mimicking the septicemic phase of the neisserial invasive diseases, we demonstrate that the presence of the MDAΦ is not associated with a higher virulence, but we show that the bacteriophage particles, by promoting bacteria-bacteria interactions, increase the biomass of bacteria colonizing a monolayer of epithelial cells. These data suggest that the increased invasiveness mediated by the MDAΦ bacteriophage is likely to be due to a better ability of the bacteria to colonize the nasopharyngeal mucosa.

## Introduction

*Neisseria meningitidis* (*Nm*) is a commensal bacterium commonly carried asymptomatically in the human nasopharynx. In a small proportion of colonized people, the bacteria invade the bloodstream from where they cause septicaemia and/or meningitis after crossing the blood brain barrier. Most meningococcal diseases are caused by bacteria belonging to only a few of the phylogenetic groups that constitute the population structure of this genetically variable organism [1]. Numerous virulence factors are expressed by meningococci. The capsular polysaccharide, the iron chelation systems [2] and the factor H binding protein are required by the bacteria to survive in the extra cellular fluids [3]. The type IV pili and Opa proteins are important for bacterial host cell interaction and allow nasopharyngeal colonization [4]. When bacteria are encapsulated, type IV pili are the sole bacterial attribute able to aggregate bacteria and to initiate the interaction with host cells. None of these virulence factors is specific of disease isolates and these bacterial attributes are also found in bacteria belonging to clonal complexes associated with a carrier state.

In order to get insights into the genetic basis responsible for the differences in pathogenic potential, a whole genome comparison using a collection of meningococci of defined pathogenic potential was performed. This study brought to light a sequence of 8 kb, designated MDA for Meningococcal Disease Associated island, which is associated with an increase ability of invasive disease [5, 6]. Subsequent studies have demonstrated that the MDA island encodes a functional filamentous prophage, designated MDAΦ, able to produce infectious filamentous phage particles [7] (S1 Fig highlights the organization of the MDAΦ genome). However the mechanism by which the MDAΦ prophage increases bacterial invasiveness remains unknown.

Horizontally transferable mobile elements (plasmids, transposons, genetics islands and bacteriophages) are responsible for the acquisition of novel properties by bacteria, such as antibiotic resistances, detoxification of heavy metals, or virulence factors [8, 9]. Filamentous bacteriophages are part of these horizontally mobile elements [10]. CTXΦ of *Vibrio cholerae*, which encodes the cholera toxin, can transduce non-toxigenic strains into toxigenic strains, contributing to the emergence of new pathogenic *V*. *cholera* clones. The Pf bacteriophages of *Pseudomonas aeruginosa* are involved in the formation of biofilm by inducing cell death and the subsequent release of bacterial DNA [11]. Moreover, the Pf bacteriophages inside the *Pseudomonas* biofilm on acellular surfaces interact with the extracellular matrix and enhance biofilm formation by increasing adhesion and tolerance to desiccation and antibiotics [12]. Recently, Secor and colleagues have shown that Pf4 bacteriophages of *P*. *aeruginosa* promote bacterial adhesion to mucine and reduce the inflammatory response [13]. Other effects of filamentous bacteriophages include horizontal gene transfer (VPIΦ of *V*. *cholerae*), increase of motility (RSS1Φ of *Ralstonia solanacearum*, SW1Φ of *Shewanella piezotolerans*) and formation of host morphotypic variants (Cf1tΦ of *Xanthomonas campestris*, Pf4Φ and Pf6Φ of *P*. *aeruginosa*) [10].

In this work we demonstrate that the presence of the MDAΦ prophage in meningococci is not associated with virulence during the septicemic phase of the disease. On the other hand, we show that phage particles increase colonization of encapsulated bacteria onto epithelial cells. Our data suggest that this effect is mediated by a large production of phage particles within the biomass of colonizing bacteria that promote bacterial aggregation via the formation of bundles of phage filaments. We propose that the production of MDAΦ phage particles increases the occurrence of disease by promoting bacterial colonization in the nasopharynx.

## Results

### MDAΦ plays no role in the septicemic phase of meningococcal disease

As mentioned above, the presence of the MDAΦ prophage in the genome of a *Nm* strain is associated with increased invasiveness [5, 6]. We aimed at determining whether its presence could increase the virulence during the septicemic phase of meningococcemia. We used a previously described experimental model of meningococcemia [14] and compared the course of infection of a wild type (WT) strain with that of an isogenic MDAΦ deleted variant. This model uses SCID mice grafted with human skin. The vascularisation inside the human skin remains of human origin even though it connects with the mice vessels. This model addresses the two events associated with the clinical presentation of meningococcemia, i.e. (i) the growth in the bloodstream and the extra cellular fluids, and (ii) the interaction with the microvessels, responsible for the thrombotic/leakage syndrome and the meningeal invasion.

Grafted-mice were injected IV with either the WT strain or an isogenic derivative deleted of the MDAΦ prophage (ΔMDA), as described in the material and methods section. Results, reported S2A Fig, did not show any significant difference in the course of infection induced by the two strains. We then performed competition experiments by infecting intravenously three grafted-mice with an equal quantity of the WT strain and the ΔMDA strain (S2B Fig). The number of bacteria in the bloodstream was determined at 1 and 18 hours after infection and the number of bacteria colonizing the graft at 18 hours [14]. The latter is directly correlated with the ability of the bacteria to interact *in vivo* with endothelial cells. The competitive index was calculated as described in the material and methods section. In all cases, the competitive index was close to one and no statistical difference was observed.

Since the ability to resist human complement is not addressed in the above mouse model and considering that one of the phage encoded protein, MDAORF6, has recently been implicated in the resistance to normal human serum when expressed simultaneously with other homologous proteins [15], we compared the ability of the WT strain and that of the ΔMDA strain to resist to complement containing human serum. The number of surviving bacteria after 30 min of contact with 60% of human serum was determined. Control experiments using heat-inactivated human serum and an isogenic non-capsulated strain were performed. The deleted MDA mutant was as resistant as the WT strain to complement containing human serum (S3 Fig). This result is consistent with the previously published results [15] which showed that an effect on the complement resistance was observed only when all homologous proteins of MDAORF6 were simultaneously deleted. Altogether these results ruled out a role of the MDAΦ prophage in the virulence of strain Z5463 during the septicemic phase of meningococcal infection.

### The presence of the MDAΦ prophage increases *Nm* colonization onto epithelial cells under flow conditions

**Fig 1:**
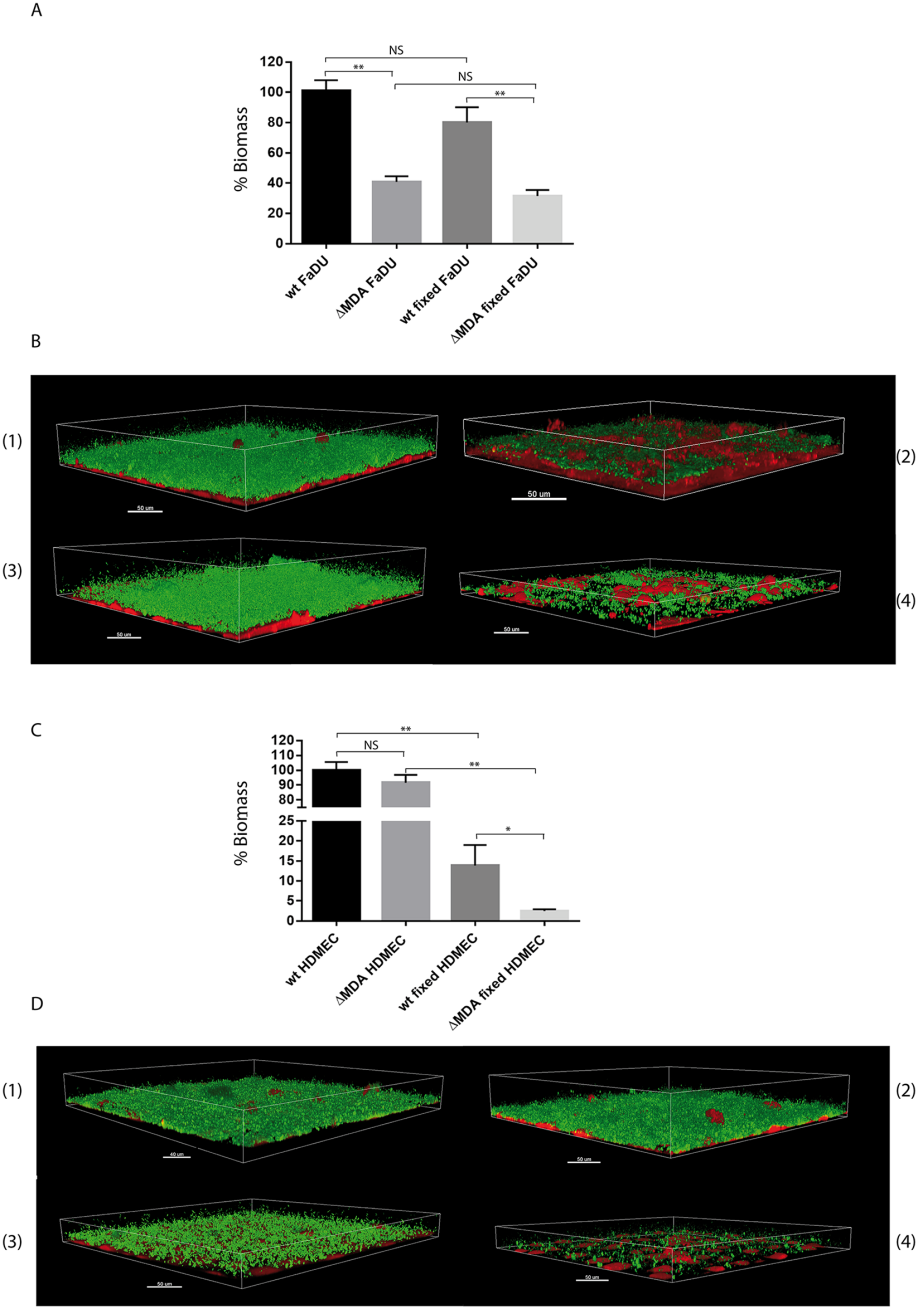
Colonization of cells by the wild type and prophage deleted strains. Quantification of the biomass on living and paraformaldehyde treated epithelial cells (FaDu). Three-dimensional reconstruction of the biomass in (A) with the Imaris software. (1) Z5463*gfp* (WT strain) on FaDu, (2) Z5463*gfp*ΔMDA on FaDu, (3) Z5463*gfp* (WT strain) on fixed FaDu, (4) Z5463*gfp*ΔMDA on fixed FaDu. Bacteria are shown in green and epithelial cells in red. Quantification of the biomass on living and paraformaldehyde treated endothelial cells (HDMEC). Three-dimensional reconstruction of the biomass in (C) with the Imaris software. (1) Z5463*gfp* (WT strain) on HDMEC, (2) Z5463*gfp*ΔMDA on HDMEC, (3) Z5463*gfp* (WT strain) on fixed HDMEC, (4) Z5463*gfp*ΔMDA on fixed HDMEC. Bacteria are shown in green and epithelial cells in red. Wild-type (Z5463*gfp*) and Z5463*gfp*ΔMDA strains were grown onto cell monolayers for 18 hours under constant flow. The biomass was quantified using the COMSTAT software. At least three independent experiments were performed. The results are normalized as the percentage of the mean of the biomass of the wild-type strain on living cells, which was set to 100%. Error bars indicate the standard errors of the mean (SEM). **p < 0.001, *p < 0.05 (Student t test), NS: not significant *p* value.

Considering the above results, we hypothesized that the presence of the phage does not confer an advantage to bacteria during the septicemic phase of the disease but in the nasopharynx. A large number of meningococci in this location may be responsible for a higher translocation rate of bacteria in the bloodstream and/or a better dissemination of the bacteria among a population, which in turn increases the number of meningococcal diseases by amplifying the number of carriers. To test this hypothesis, we assessed the ability of the WT strain and that of the MDA deleted isogenic strain to interact with a monolayer an epithelial cell line derived from a pharyngeal tumor, the FaDu cells. Initial experiments were performed during a short period of time, and results, reported S4 Fig, did not show any difference between two isogenic isolates, carrying or not a MDAΦ prophage. Considering that, in the nasopharynx, the site-of-entry of meningococci, adherent bacteria are subject to a flow, due to the presence of ciliated cells [16], we assessed the ability of bacteria to colonize a monolayer during a long period of time (18 hours) under constant flow in order to be closer to the *in vivo* situation [17]. Two isogenic fluorescent strains, Z5463*gfp* and Z5463*gfp*ΔMDA were used to quantify the biomass of bacteria adhering onto the monolayer (see Material and methods). Results are shown Fig 1A and 1B. The biomass covering the monolayers formed by the MDAΦ deleted strain was constantly reduced by 40 to 50% when compared to that of the parental strain. It should be pointed out that this difference was not explained by a difference in growth rate of the two strains as the doubling time of these strains was identical (S5 Fig). It should be pointed out that a similar phenotype was observed using another epithelial cell line, a monolayer of Calu-3 cells (cells from a lung adenocarcinoma) (S6 Fig).

The above results showing an increased colonization onto epithelial cells of the isolate containing an MDAΦ prophage were surprising in light of the *in vivo* data that did not show a selective advantage of the MDAΦ producing strain inside the skin graft where bacteria interact with endothelial cells. We subsequently determined the ability of the phage to promote bacterial colonization onto a monolayer of endothelial cells using the same conditions as above for epithelial cells. As shown Fig 1C and 1D, the WT and MDAΦ deleted strains showed the same level of colonization onto endothelial cells. A possible explanation for this discrepancy observed with the two cell types was the different cross talk observed when meningococci infect endothelial and epithelial cells, as this has been previously suggested [18]. To test this hypothesis, experiments were also performed using fixed monolayers (*i*.*e*. pretreated with a solution of 4% paraformaldehyde). As shown Fig 1C, the WT strain colonized significantly less a monolayer of fixed endothelial cells when compared to that of living cells. On the other hand, colonization of the MDAΦ deleted strain was dramatically reduced onto fixed cells when compared to that of the WT strain. In contrast, data obtained on a monolayer of fixed FaDu epithelial cells were similar to those obtained on living cells (Fig 1A and 1B). Altogether these results are consistent with the *in vivo* data which did not show any advantage to prophage containing strains when adhering onto the microvessels of the skin graft and clearly showed that the ability of the prophage to increase bacterial colonization is specific of epithelial cells and depends upon the bacteria host cell cross talk.

### Phage particles are responsible for the enhancement of bacterial colonization onto epithelial cells

We next assessed whether the above phenotype observed onto FaDu cells was a consequence of the production of phage particles or a consequence of the presence of prophage encoded genes. Experiments were performed using strains deleted in two genes that have been shown to be important for phage replication and the production of viruses, *MDAorf1* and *MDAorf9* (S1 Fig) [5, 7]. Strains Z5463*gfp*Δ*orf1* and Z5463*gfp*Δ*orf9* described in the material and methods section are unable to produce replicative cytoplasmic forms of the phage, and infectious particle, even though they carry a prophage in their genome. As shown S7 Fig, these strains colonized a monolayer of FaDu cells at a level identical to that of a prophage deleted strain. Altogether these results are in favour of a direct role of the virus particles in the observed phenotype.

**Fig 2:**
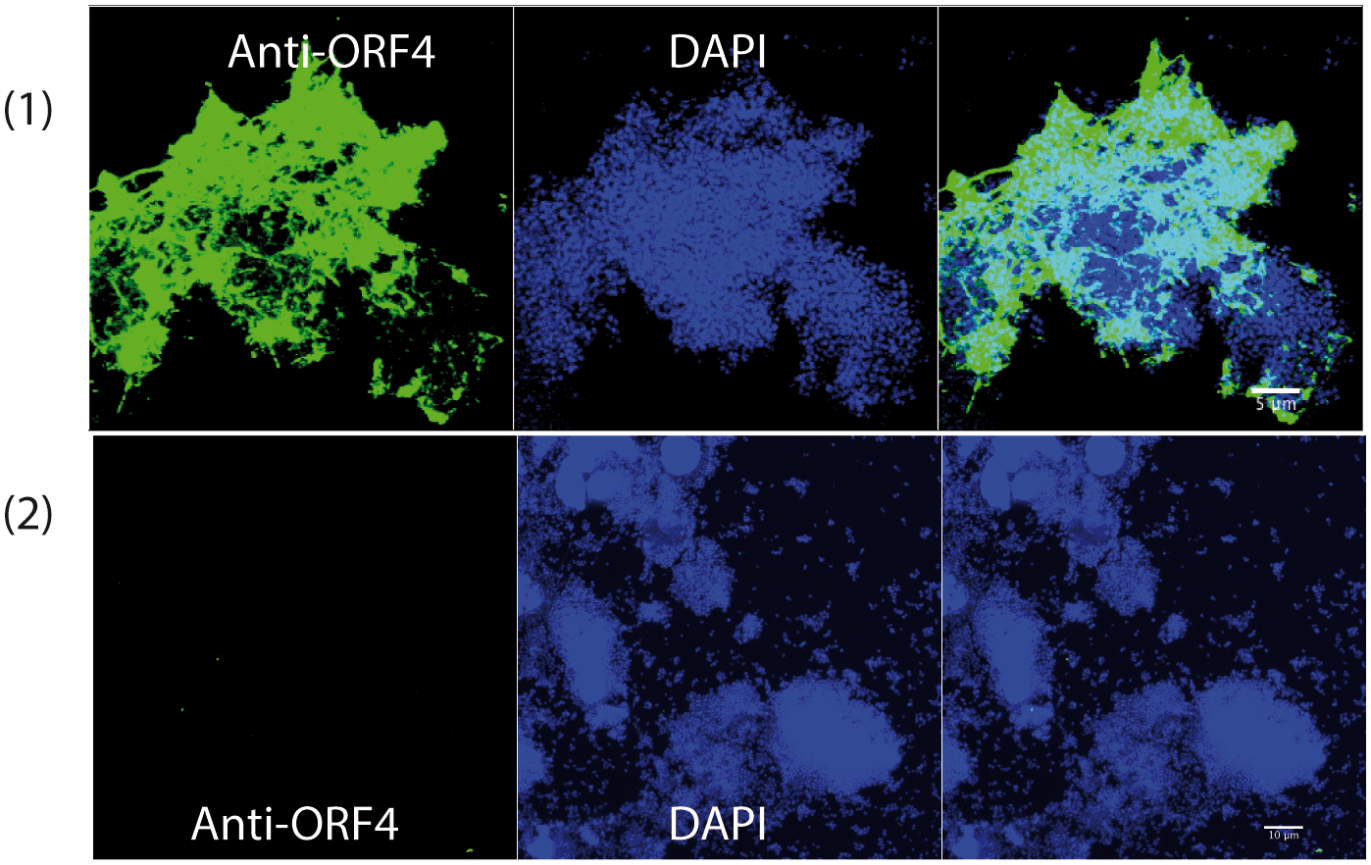
Quantification of MDAΦ in the biomass. Immunofluorescence microscopy performed on the biomass of bacteria colonizing FaDu cells. Bacteria grown under constant flow were harvested by aspiration from the monolayer of epithelial cells, as described in the material and methods section. Bacteriophages were labelled (in green) using the polyclonal antibody anti-MDAORF4 directed against the major capsid protein of MDAΦ. Bacteria are stained with DAPI (in blue). (1) Z5463, the green staining which correspond to the bacteriophages represents 75% of the DAPI surface; (2) Z5463ΔMDA, the green surface is only 0.24% of the DAPI surface.

Consistent with the above results, was the intense labelling of the major capsid protein, MDAORF4, inside the biomass covering the monolayer of cells (Fig 2A). This suggested that phage production occurred during bacterial colonization of the epithelial cells. To confirm this point, phage DNA and phage proteins were monitored during bacterial colonization of the epithelial monolayer. The quantity of circular MDAΦ DNA per chromosome increases as a function of time (Table 1), and consistently the amount of MDAORF10 and MDAORF5 proteins (S8 Fig) increased in a proportion higher than expected from the growth of the biomass (see panel C S8 Fig). Considering the above results showing the absence of phenotype onto endothelial cells, we aimed at precising the phage production onto this cell type. The quantification of the circular MDAΦ DNA per chromosome in the biomass covering endothelial cells reveals the absence of production of MDAΦ during the formation of the biomass under flow on endothelial cells (Table 1). On the other hand onto fixed endothelial cells, the quantity of circular MDAΦ DNA per chromosome increased significantly (Table 1). Altogether, these results are consistent with the above reported data showing that the prophage does not provide a selective advantage to bacteria colonizing endothelial cells.

**Table 1:** Quantification by real time PCR of the number of MDAΦ DNA per bacterial chromosome.

		Number of MDAΦ per bacterial chromosome *	+/-SEM	*p* value ^a^
Inoculum		4.8	0.67	
FaDu	22h	25.0	9.02	**<0.001**
HDMEC	22h	4.3	0.51	0.602
Fixed HDMEC	22h	9.1	0.67	**<0.001**

* extrapolated per copy of the *pgm* gene

^a^ one-way ANOVA test (compared to the value obtained with the inoculum)

Altogether these results suggest that the increased colonization observed during interaction onto epithelial cells was associated with the production of viruses.

### MDAϕ is responsible for the formation of bacteria-bacteria interactions

**Fig 3:**
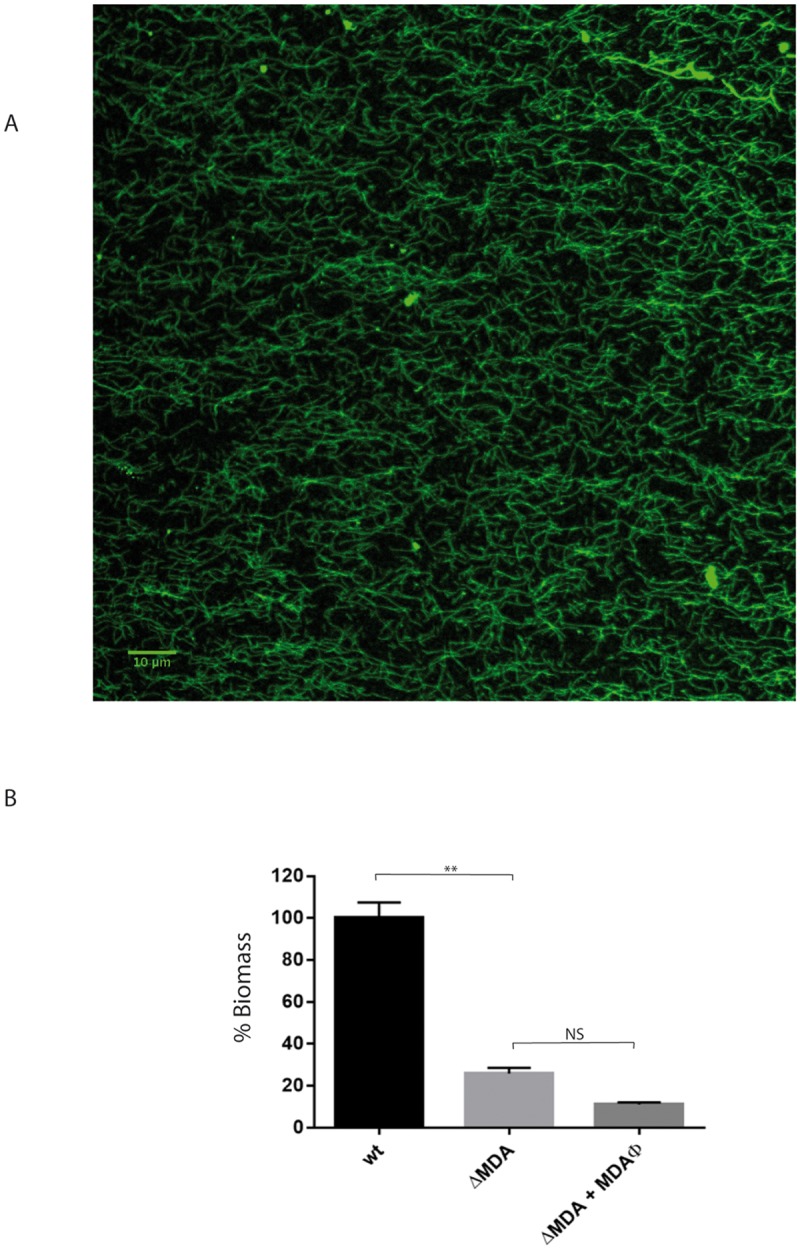
Addition of exogenous bacteriophages. **A.** Immunofluorescence microscopy performed on the phage preparation using the polyclonal antibody anti-MDAORF4 directed against the major capsid protein of MDA. **B.** Z5463*gfp* and Z5463*gfp*ΔMDA strains were grown on FaDu epithelial cells for 18 hours under constant flow. In one well, MDAΦ was added to the growth of the prophage deleted strain during the entire experiment at a final concentration of 10^12^ particles/mL. At least three independent experiments were performed. The results are normalized as a percentage of the mean biomass of the wild-type strain, which was set to 100%. Error bars indicate the standard errors of the mean (SEM). **p < 0.001 (One-way ANOVA), NS: not significant *p* value.

We next aimed at determining the mechanism by which the production of MDAΦ increases the biomass of bacteria onto epithelial cells. We first tested the hypothesis that the addition of exogenous bacteriophages during bacterial adhesion to a culture of a phage-deleted strain could mimic the phenotype observed. Bacteriophages were prepared as described in the material and methods section, and the ability of strain Z5463*gfp*ΔMDA to form a biomass on cells was determined in the presence of 10^12^ bacteriophages per mL in the supernatant. Results are presented Fig 3. No significant difference was observed with or without the presence of exogenous bacteriophages, this strongly suggests that the phage has to be produced locally by the bacteria to increase the formation of the biomass.

**Fig 4:**
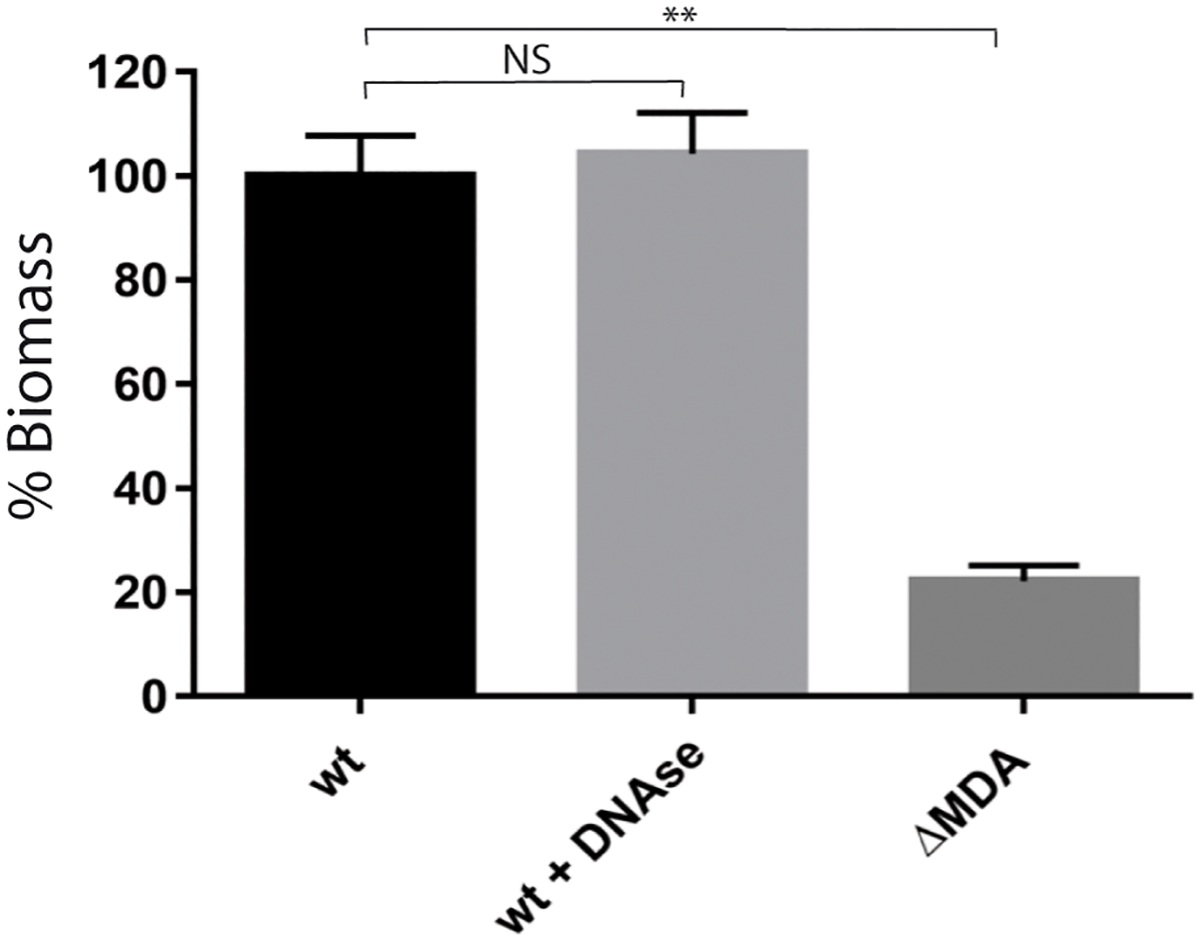
DNAse and formation of the biomass colonizing epithelial cells. Z5463*gfp* and Z5463*gfp*ΔMDA strains were grown in the presence on FaDu epithelial cells for 18 hours under constant flow. To assess the potential role of extra cellular DNA (eDNA), experiments were carried in the presence of DNAse at a concentration of 1 μg/mL. At least three independent experiments were performed. The results are normalized as a percentage of the mean biomass of the wild-type strain, which was set to 100%. Error bars indicate the standard errors of the mean (SEM). **p < 0.0001 (One-way ANOVA), NS: not significant *p* value.

The production of a phage by a bacterial population is possibly associated with bacterial lysis and subsequent release of extra cellular DNA (eDNA) that in turn can participate in the formation of a biofilm [19]. Even though this hypothesis was unlikely considering that filamentous phages such as MDAΦ do not have a lytic cycle, we ruled out a possible role of extra cellular DNA (eDNA) by quantifying the biomass covering the monolayer after having added DNAse in the culture media to degrade possible eDNA in the extra cellular matrix. The final concentration of DNAse was 1 μg/mL corresponding to that routinely used to degrade eDNA in extra cellular matrix [20]. As shown Fig 4, the biomass of colonizing bacteria was identical regardless of the presence of DNAse.

**Fig 5:**
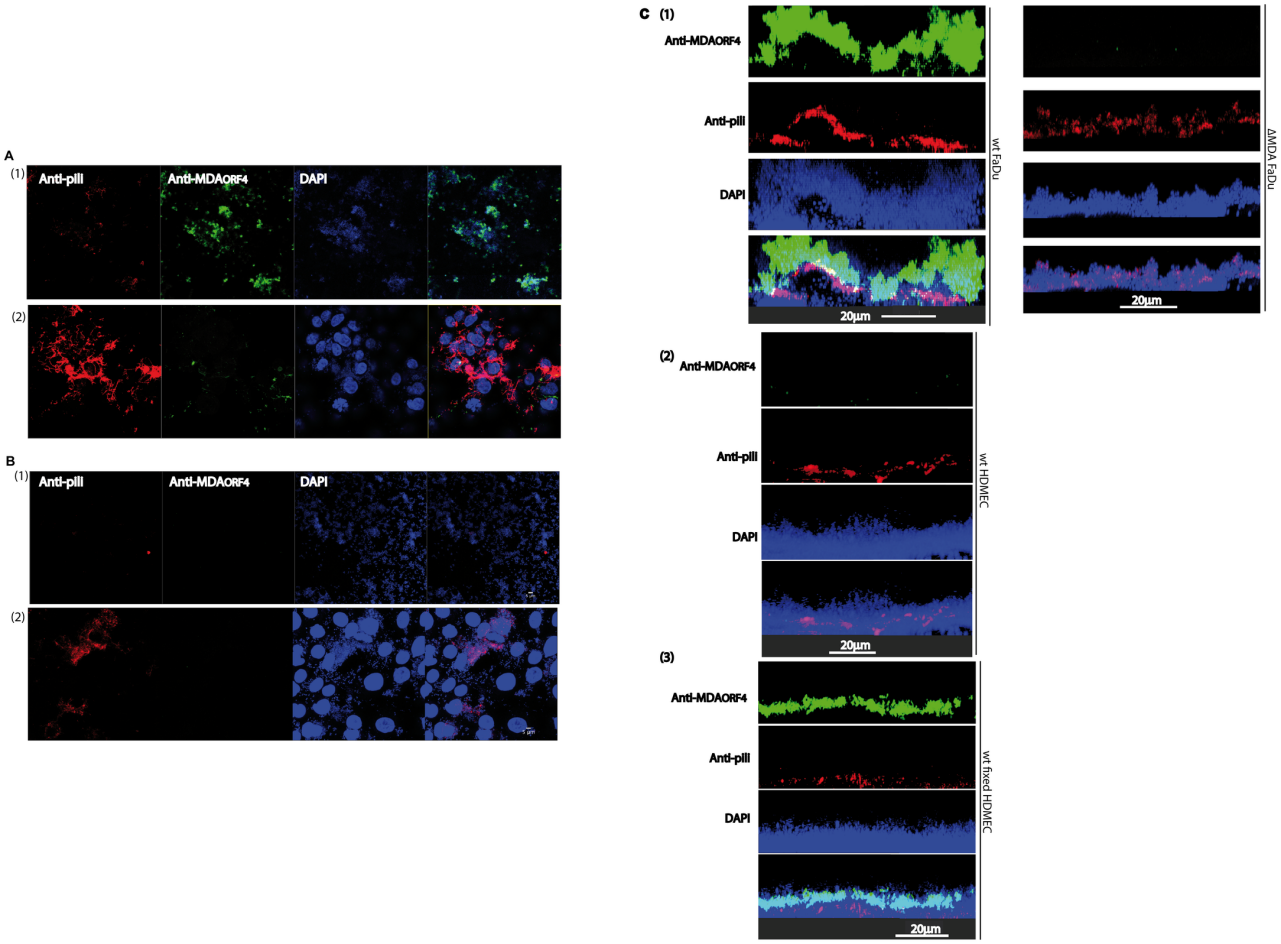
Detection of phage particles and type IV pili in bacteria colonizing the epithelial cell monolayer. Monolayers of FaDu cells in a flow chamber were infected either with the wild-type strain Z5463(SB-*aph3’*) (A) or with the MDAΦ deleted strain (Z5463(SB-*aph3’*)ΔMDA) (B) under constant flow for 18 hours. Bacteria were then aspirated from the flow chamber leaving bacteria adhering onto the apical surface of the epithelial cells. The material recovered by aspiration ((1) top panel) and the remaining bacteria adhering onto the monolayers ((2) bottom panel) were stained by immunofluorescence using the anti MDAORF4 polyclonal antibody (in green) and the 20D9 monoclonal antibody (in red). With both strains, bacteria that have not been harvested are covering the host cells. These bacteria are heavily piliated, and in the case of the WT strain very few phage particles were detected on this material, the green staining which corresponds to bacteriophages represents less 6% of the red surface which correspond to the pili staining. On the other hand, in the material harvested by aspiration corresponding to the upper layers of the biomass, bacteria are surrounded by large quantity of bacteriophages (the red staining which corresponds to the pili represents 25% of the green surface which corresponds to the phage). (C) (1) Left pane, XZ section of a monolayer of FaDu cells in a flow chamber infected with the wild-type strain Z5463(SB-*aph3’*) for 18 hours. Right panel XZ section of a monolayer of FaDu cells in a flow chamber infected with the MDAΦ deleted strain (Z5463(SB-*aph3’*)ΔMDA) for 18 hours. (2) XZ section of a monolayer of HDMEC cells in a flow chamber infected with the wild-type strain Z5463(SB-*aph3’*) for 18 hours. (3) XZ section of a fixed monolayer of HDMEC cells in a flow chamber infected with the wild-type strain Z5463(SB-*aph3’*) for 18 hours. The biomass was stained by immunofluorescence using the anti-MDAORF4 polyclonal antibody (in green), the 20D9 monoclonal antibody (in red) and DAPI in blue.

Type IV pili have long been identified as being the main bacterial attribute promoting both the formation of bacterial aggregates and the initial interaction of encapsulated meningococci onto host cells [21]. Considering the above results suggesting a direct role of viral particles, we determined the localisation of both pili and bacteriophages inside the biomass. To be able to visualize both, MDAΦ and pili, we used a derivative of strain Z5463 which was modified in order to express a pilin variant from strain 2C4.3, designated SB. The pili encoded by this variant can be labelled by a monoclonal antibody, 20D9. The construction of this strain designated Z5463(SB-*aph3’*) has been previously reported [7]. Bacteria colonizing a FaDu epithelial cell monolayer in a laminar flow chamber were harvested by aspiration as described in the material and methods section. This step removed most of the bacteria colonizing the epithelial cells, leaving only bacteria strongly adhering to the epithelial monolayer. Both adhering bacteria and bacteria peeled off the epithelial cells were labelled using the 20D9 monoclonal antibody and a polyclonal antibody directed against the MDAORF4, the major capsid protein [7]. As shown Fig 5A, bacteria obtained from the aspiration were heavily labelled by the polyclonal antibody against the major phage capsid protein, but almost no labelling was obtained using the 20D9 anti-pili monoclonal antibody. In contrast, bacteria still interacting with the epithelial monolayer were piliated but were not associated with bacteriophages. A similar experiment was performed using a prophage-deleted isogenic derivative. Only bacteria adhering strongly to epithelial cells were piliated whereas those peeled off the monolayer were not piliated (Fig 5B). These results suggested that bacteria close to the apical surface of the epithelial monolayer were piliated but not surrounded by bacteriophages whereas those located in the upper layers of the biomass produced large amount of bacteriophages but were not piliated. This finding was confirmed by performing XZ sections of an infected epithelial monolayer. Results are shown Fig 5C. After 18 hours of interaction onto a monolayer of epithelial cells (i) piliated bacteria are found close to the apical surface of the epithelial cells, in these layers very little labelling of bacteriophages is visible, and (ii) in the upper layers, bacteria are non piliated but surrounded by large amount of bacteriophages. It should be pointed out that similar XZ sections performed on infected living endothelial cells revealed the absence of MDAΦ particles at the surface of the biomass (Fig 5C). On the other hand, onto fixed endothelial cells, the XZ sections confirmed a large production of MDAΦ particles in the upper layers of the biomass (Fig 5C). Regardless of the cell type studied, the presence of type 4 pili is always restricted to bacteria associated with the apical surface of cells. These results are consistent with the data reported above showing that the prophage does not provide a selective advantage to bacteria colonizing endothelial cells.

**Fig 6:**
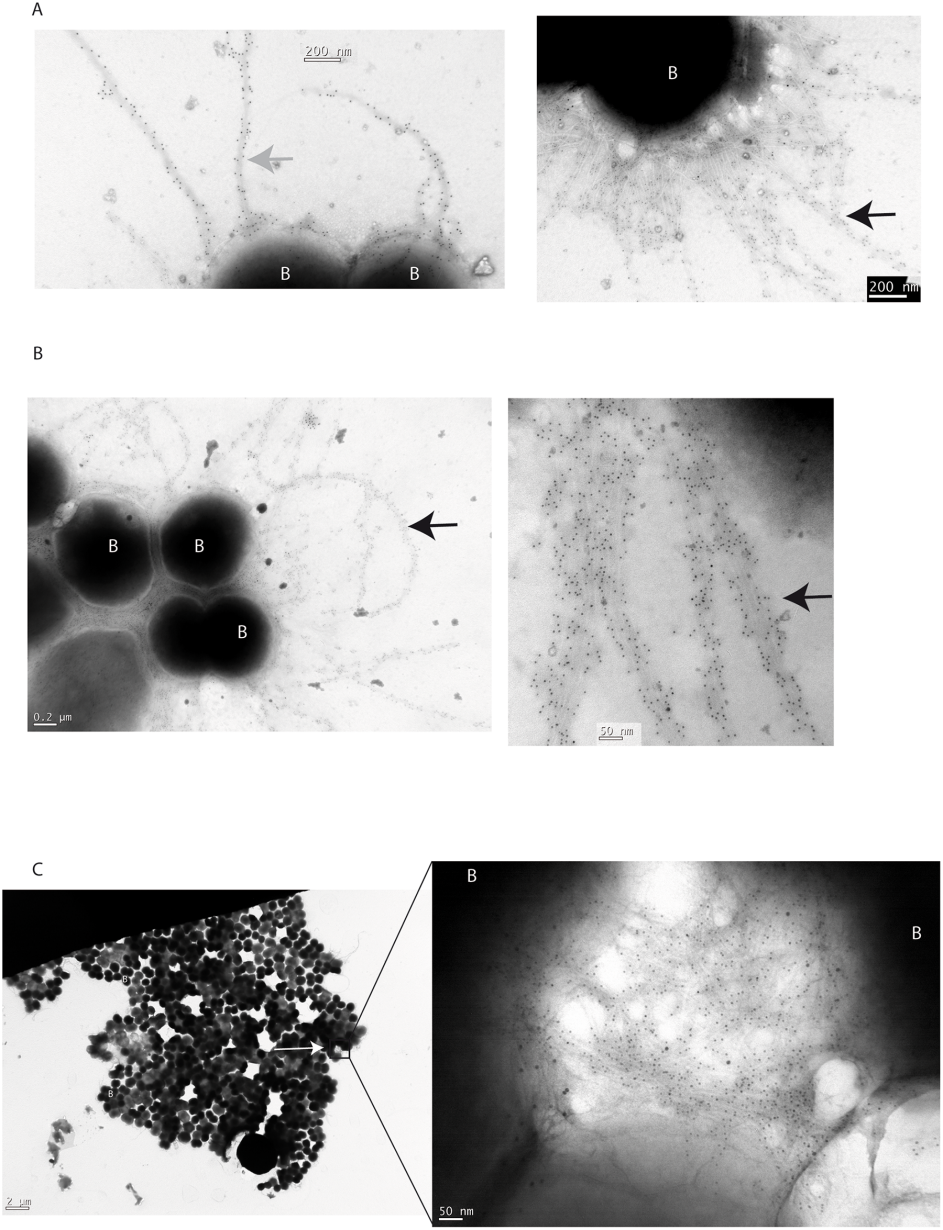
Immunogold-labelling of MDAΦ and type IV pili of material harvested from a flow chamber by aspiration. Two antibodies were used: (i) the anti-MDAORF4 polyclonal antibody labelled with 8 nm-diameter gold particles and (ii) the monoclonal antibody 20D9 directed against the SB pilin variant and labelled with 12 nm-diameter gold particles. (A) Positive control, strain Z5463(SB-*aph3’*) grown under standard conditions. Left panel, bacteria are labelled by 12 nm beads corresponding to pili, the gray arrow points toward 12 nm beads. Right panel, bacteria surrounded only by filaments labelled by small particles corresponding to filamentous bacteriophages, the black arrow points toward 8 nm beads. (B) Material harvested from the biomass covering a monolayer of FaDu cells infected by Z5463(SB-*aph3’*) and labelled by the above two antibodies. Most of the material surrounding the bacteria are labelled by 8 nm particles and therefore correspond to filamentous bacteriophages (black arrow). No labelling with 12 nm beads corresponding to type IV pili was detected. (C) Global view of bacteria harvested from a flow chamber with enlargements. Only phage filaments are detected. Magnification is noted. Letters B in white label bacteria.

Electron microscopy combined with immunogold labelling using both the anti-pilin 20D9 monoclonal antibody and anti-MDAORF4 polyclonal antibody were performed on bacteria obtained as above by aspiration. Results are reported Fig 6. Images shown Fig 6A confirmed that, using these labellings, we could visualise independently type IV pili and bacteriophages surrounding bacteria. It should be pointed out that the morphology of the bacteriophages is indistinguishable from that of pili. Numerous bacteriophage filaments remained linked to the cell wall. This is consistent with the fact that being a filamentous phage, the release of MDAΦ is not a consequence of bacterial lysis but the consequence of a secretion through the outer-membrane. The labelling of the aspirated biomass confirmed that in the upper part of the biofilm most of the meningococci were producing bacterial bacteriophages and not type IV pili (Fig 6B). In addition they showed (i) that numerous viruses remained associated with the bacterial cell wall, and (ii) that phage-phage interactions occurred leading to the formation of bundles of viruses that are able to connect bacteria (Fig 6B and 6C). The ability of this phage to bundle was confirmed on an immunofluorescence staining of a phage preparation using the anti MDAORF4 polyclonal antibody that showed that MDAΦ phage particles are able to form large bundles of filaments (Fig 3A). Altogether these data suggest that, in the upper layers of the biomass colonizing the biofilm, numerous bundles of viruses embed bacteria (Figs 5C, 6B and 6C) and that these bundles by interacting with phage particles still associated with the cell wall increase bacteria/bacteria connections (Fig 6C). The lack of complementation by exogenous phage suggests that the phenotype observed is specific of phage producing bacteria.

**Fig 7:**
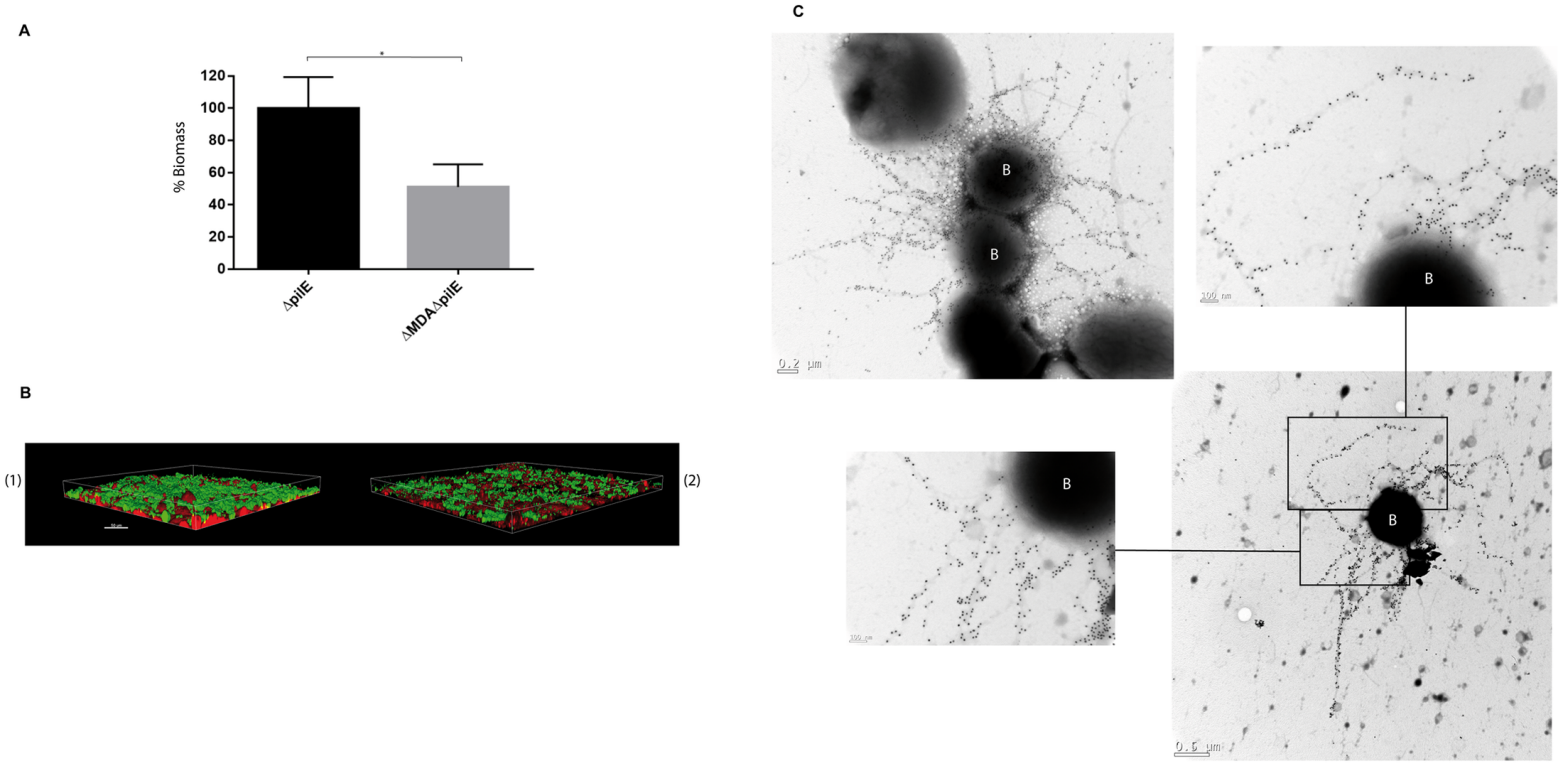
Meningococcal biofilm formation of non piliated strains onto epithelial cells. (A) Quantification of the biomass covering cells infected with *pilE* mutants (Z5463*gfp*Δ*pilE* and Z5463*gfp*Δ*pilE*ΔMDA). Mutants were grown onto FaDu epithelial cells for 18 hours under constant flow. At least three independent experiments were performed. The results are normalized as a percentage of the mean biomass of Z5463*gfp*Δ*pilE*, which was set to 100%. Error bars indicate the standard errors of the mean (SEM). *p < 0.05 (Student t test). (B) Three-dimensional biomass of colonizing bacteria reconstructed with the Imaris software. (1) non piliated strain. (2) a prophage deleted isogenic mutant of the non piliated strain. Both strains were grown onto FaDu epithelial cells for 18 hours under constant flow shear stress. Representative images of experiments performed on at least three different occasions. Bacteria are stained with GFP (in green) and epithelial cells are stained with cytoplasmic Cell Tracker Orange CMTMR (in red). (C) Immunogold-labelling of Z5463*gfp*Δ*pilE* strain. Material was harvested from a flow chamber as above and labelled with the anti-MDAORF4 polyclonal antibody. In this experiment, only 18 nm-diameter gold particles were used. Global view with some enlargements. The letter B in white corresponds to bacteria.

Similar experiments were performed, as described in the material and methods section, with *pilE* derivatives of strains Z5463*gfp* and Z5463*gfp*ΔMDA. Type IV pili is the main bacterial attribute allowing the interaction of encapsulated meningococci with host cells, and as expected, very few non-piliated derivatives of strain Z5463*gfp*ΔMDA were interacting with the monolayer. On the other hand a larger biomass of the non piliated derivative of strain Z5463*gfp* which carries the prophage, was colonizing the monolayer of host cells, even though the thickness of the biomass was reduced compared to that of a wild type piliated strain (Fig 7A and 7B). Electron microscopy performed on the harvested biofilm confirmed that the prophage containing strain was able to produce large amount of phage particles that can remain associated to the bacterial cell wall, and provide interbacterial connection via phage/phage interactions (Fig 7C).

## Discussion

The presence of the bacteriophage MDAΦ has been associated with hypervirulent clonal complexes of *N*. *meningitidis* [5],[6]. The initial hypothesis suggested the presence of virulence factors encoded by the prophage giving a selective advantage during the bloodstream phase of the disease. Our data obtained with our animal model mimicking the septicemic phase of the neisserial invasive diseases do not support this hypothesis. On the other hand, our results suggest that the virulence factor encoded by the prophage is the phage particle itself promoting bacterial aggregation when the bacteria interact with epithelial cells. The production of phage in the nasopharynx is therefore likely to increase the biomass of bacteria at the site of entry. This increased biomass could in turn increase the frequency of bacterial dissemination in the bloodstream and/or the dissemination of the bacteria inside a population, which, by increasing the number of carriers, is responsible for a higher rate of diseases. These hypothesis are consistent with previous results reported for *Streptococcus pneumonia*e, where an association between an increased density of nasopharyngeal colonization has been associated with a higher rate of invasive pneumococcal pneumonia [22].

**Fig 8:**
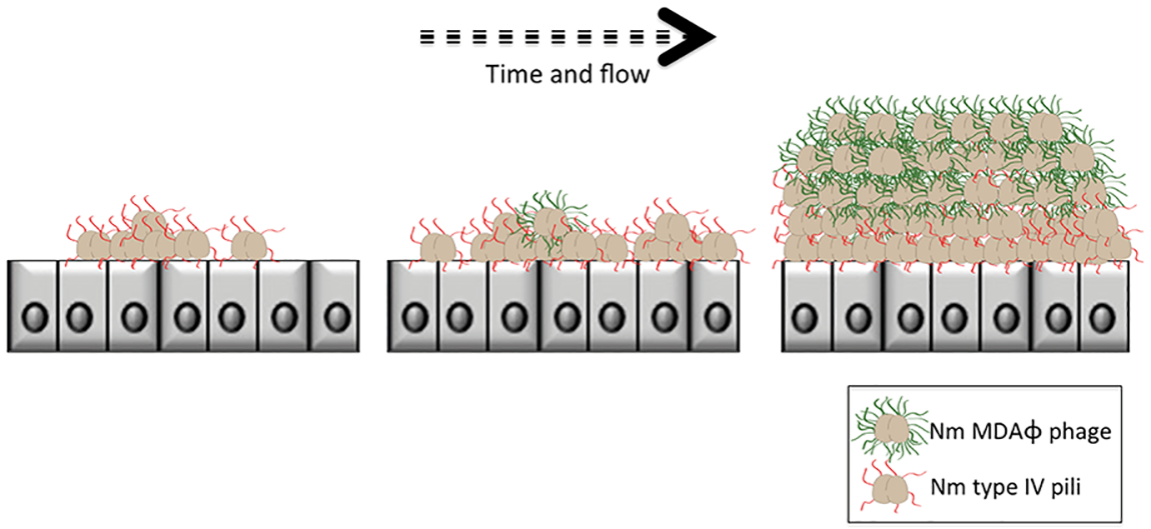
Proposed model for the role of MDAΦ bacteriophage in the commensal lifecycle of *Nm*. Type IV pili are represented in red and bacteriophage in green. After the initial adhesion mediated by type IV pili, bacteria multiply and loose piliation. On the other hand, bacteria in direct contact with the host cells remained piliated. Phage production is activated in the upper layers of the biomass and bacteriophages, which remain in part attached to the bacterial surface, form bundles which stabilize bacteria/bacteria interactions.

Capsulated meningococci interact with epithelial cells *via* their type IV pili. Following this initial interaction, bacteria divide and, as previously reported, repress the transcription of the major pilin subunit PilE [23], retract their pili, and loose piliation [24], leading to the formation of layers of piliated bacteria directly in contact with the apical surface of the host cells, and bacteria loosing their pili are washed away from the monolayers by the shear stress. These previous reports are consistent with the data shown with the MDAΦ deleted strain. On the other hand, with the WT parental strain the biomass is thicker than expected, as bacteria in the upper layers are non piliated and included in a network of MDA phage particles which form large bundles able to connect bacteria most likely via interactions with filamentous bacteriophages remaining associated with the outer membrane of bacterial cells as they extrude through the cell wall (Fig 8). The mechanism responsible for the segregation of phage producing strains could resemble the one identified in *Neisseria gonorrhoeae* for mixed population of piliated and unpiliated bacteria [25], where non-piliated bacteria segregate from those that are piliated. The authors suggested that this cell-sorting was under the control of active forces which rely on similar physical principles as those observed in developing embryos.

A striking observation is the fact that this effect of the phage on bacteria colonization is specific of epithelial cells. Indeed, the prophage does not provide a selective advantage for bacterial colonization onto endothelial cells. Meningococci induce signalling pathways on epithelial and endothelial cells that are known to be different [18]. On endothelial cells, adhesion of *N*. *meningitidis* leads to the recruitment of the junctional components [26] and to the formation of microvilli like structures which allow bacterial protection from shear stress [27]. We hypothesized that the cross talk between bacteria and endothelial cells could be responsible for our observation, the fact that fixed endothelial cells behave like epithelial cells support this hypothesis. The molecular mechanisms responsible for this are unknown, a possible explanation is to consider that the formation of microvilli on the apical surface of endothelial cells by protecting the bacteria from shear stress prevent the production of phage particles, which may not be the case onto epithelial cells.

Interestingly, Sigurlàsdòttir and colleagues have shown that, after initial adhesion on epithelial cells, lactates, produced by host cells, initiate rapid dispersal of microcolonies of *N*. *meningitidis* [28]. In our model of adhesion under flow conditions, the permanent renewal of the medium prevents the increase of lactates concentration and the subsequent dispersal of bacteria.

Another striking result is the fact that meningococci seem to produce either pili or bacteriophages. Indeed, type IV pili and bacteriophages labelled very rarely the same bacterium. This is somewhat consistent with previous results showing that some of the type IV pilus machinery is used by the phage for its secretion, especially the secretin PilQ which is used to extrude the pilus fiber and the phage filament through the outer membrane [5]. This suggests that production of pili and bacteriophages are co-regulated and mutually exclusive. Indeed as already mentioned following initial adhesion, piliation is down regulated by the inhibition of transcription of the major pilin subunit and pilus retraction [23, 24, 29]. It is likely that these regulatory pathways control the induction of bacteriophage production.

Altogether our data by demonstrating that MDAΦ increase the colonization of bacteria specifically onto epithelial cells suggest that the increase invasiveness observed by strain carrier this prophage may be a consequence of a high bacterial load at the site-of-entry which in turn increase the chance of translocation in the bloodstream and/or the dissemination of the bacteria in a population and the number of carriers.

## Materials and methods

### Bacterial strains and culture media

Z5463, formerly designated C396, is a serogroup A strain isolated from the throat of a patient with meningitis in The Gambia in 1983 (gracefully provided by J. Parkhill [30]). The genomic sequence of this strain is deposited in the PubMLST website ([Neisseria PubMLST:17882] [31]).

*Neisseria* were grown at 37°C in 5% CO_2_ on GC medium base (Difco) containing Kellogg’s supplements [32], or in GC-liquid medium [1.5% proteose peptone (Difco); 0.4% K_2_HPO_4_, 0.1% KH_2_PO_4_, 0.1% NaCl, with 12 μM FeSO_4_ and Kellog’s supplements].

Kanamycin (Km) was used at a concentration of 200 μg/mL, spectinomycin (Sp) at 75 μg/mL, erythromycin (Em) at 3 μg/mL and chloramphenicol (Cm) at 5 μg/mL.

### Serum survival

Serum survival was performed as previously described [15, 33] with minor modifications. Bacterial strains were grown overnight on GC medium base plates and then cultures in chemically defined medium (CDM) supplemented with 1 mg/mL of Cohn fraction IV from human serum (Sigma-Aldrich). The CDM was Catlin 6 medium modified to contain 5.5 mM glucose, 4 mM D,L-lactate, 50 μM cysteine and 150 μM cystine [15]. Cultures were grown until an optical density at 600nm (OD_600nm_) of 0.6. A 1/600 dilution of this broth was realized in 60% of NHS (Normal Human Serum) or hiNHS (heat-inactivated Normal Human Serum) and incubated at 37°C. Normal Human Serum AB-type (PAA Laboratories) used was handled in a manner to preserve complement activity [34] or heat-inactivated at 56°C during 30 min. The percentage of bacteria surviving at 30 min was determined. Each assay was performed in triplicate.

### Construction of mutants

The list of strains and mutants used in this study is reported in S1 Table. Mutants Z5463ΔMDA and *orf1* were previously described [5, 7].

Z5463(SB-*aph3’*) was obtained following transformation of DNA of strain *Nm* 8013 SB-*aph3’* [35] into Z5463 and selecting for Km resistance [7].

Z5463 expressing a green fluorescent protein (GFP) under IPTG-inducible promoter was obtained by transformation of plasmid pAM239 [36], the resulting strain was designated Z5463*gfp*. Z5463*gfp*ΔMDA, Z5463gfpΔ*orf1* and Z5463gfpΔ*orf9* were obtained following transformation of Z5463*gfp* with DNA of strains Z5463ΔMDA, Z5463Δ*orf1* and Z5463Δ*orf9* respectively [7].

Z5463*gfp*Δ*pilE* and Z5463*gfp*ΔMDAΔ*pilE* were obtained following transformation of the strains Z5463*gfp* and Z5463*gfp*ΔMDA by the DNA of Z5463Δ*pilE* (erythromycin resistant) described by Meyer and collaborators [7].

To engineer a non-capsulated strain, a mutant of the gene *lipA* was generated. A previously described mutation [37] was amplified by PCR and introduced into Z5463 by transformation.

### Ethics statement

The experimental procedures described in this paper were conformed to the European ethical legislation (Directive 2010/63/EU) and with the guidelines established by the French regulations (Décrets 87–848, 2001–464, 2001–486 and 2001–131). The experimental protocol was approved by the Comité d'Expérimentation Animale de l'Université Paris Descartes (France, project number CEEA 12–030).

Six weeks old CB17/Icr-Prkdcscid (Severe Combined Immunodeficiency: SCID) female mice were obtained from Charles River Laboratories (Saint Germain sur l'Arbresle, France). Human skin grafts were obtained anonymously from surgical waste from patient undergoing plastic surgery at Groupe Hospitalier Paris Saint-Joseph (Paris, France). According to the French legislation, the patients were informed of the research finality and their non-opposition was orally received.

### Infection of SCID mice grafted with human skin infection

CB17/Icr-*Prkdc*^*scid*^/IcrIcoCrl female mice were grafted with human skin as described by Join-Lambert *et al*. [14]. *Nm* strains were grown overnight at 37°C on GCB agar plates prepared without iron and supplemented with deferoxamine (Desferal, Novartis) at a final concentration of 15 μM. Bacterial colonies were harvested and cultured in RPMI with 1% bovine serum albumin medium and 0.06 μM deferoxamine with gentle agitation to reach the exponential phase of growth. Bacteria were then resuspended in physiological saline. All mice received 10 mg of human holotransferrin (R&D Systems) administered intraperitoneally just before infection.

For the single infection model, six grafted mice were infected IV with 10^7^ CFU of WT strain or the ΔMDA isogenic strain. The injected dose is just below the LD50. The numbers of CFU in blood and in the graft were determined at 1 and 18 hours.

To obtain a competitive index, three grafted mice were infected intravenously with a mix of 5.10^6^ CFU of each strain. The numbers of CFU in the blood and in the graft were determined. The number of CFU corresponding to the ΔMDA strain was obtained by determining the number of spectinomycin resistant colonies. The competitive index was calculated by the ratio of [log (UFC_Z5463ΔMDA_)/log (UFC_WT_) in the blood or in the graft] on [log(UFC_Z5463ΔMDA_)/log(UFC_WT_) of the inoculum].

### Cell culture conditions

The pharynx carcinoma-derived FaDu epithelial cell line and the lung adenocarcinoma-derived Calu-3 epithelial cell line were obtained from the American Type Culture Collection. HDMEC (Primary Human Dermal Microvascular Endothelial Cells) were purchased from Promocell. FaDu cell lines were grown in Ham F-12 medium (PAA Laboratories) supplemented with 10% fetal calf serum (FCS; PAA Laboratories), 20 mM HEPES (PAA Laboratories) and 1% penicillin-streptomycin-amphotericin (PSA; PAA Laboratories). Calu-3 cell lines were grown in Opti-MEM (Gibco) supplemented with 5% fetal calf serum (FCS; PAA Laboratories). HDMEC were grown in ECM (Endothelial Cell Medium with supplements provided by the manufacturer, Promocell), 20 mM HEPES (PAA Laboratories) and 1% penicillin-streptomycin-amphotericin (PSA; PAA Laboratories). Cells were grown at 37°C in a humidified incubator under 5% CO_2_.

The cells were fixed using a solution of PBS-4% paraformaldehyde (PFA) during 20 min.

### Phage preparation

Phage preparation was performed as previously described [7]. Bacteria were pelleted from 200 mL of an overnight culture in GC-liquid medium. After filtration at 0.45 μm, the supernatant was treated for 3 h at 20°C with DNase I and RNase A, 25 μg/mL each. Phage was precipitated by addition of 10% NaCl and 20% polyethylene glycol 6000, and overnight incubation at 4°C. The phage was then pelleted by centrifugation at 11,000 g for 30 min, and resuspended in PBS 1X and added directly to the cell medium at a final concentration of 10% during the bacterial colonization onto epithelial monolayers. The concentration of phage was determined by real time PCR using a preparation of DNA of strain Z5463Δ*orf1* as standard.

### Bacterial colonization onto epithelial and endothelial monolayers

Short-term adhesion of meningococci to FaDu cells was performed as described previously [38], with minor modifications. The 24 well plates were seeded with 10^5^ cells per well.

Before the assay, bacteria grown on GCB agar plates were adjusted to a specific OD_600nm_ and then incubated for 2 h at 37°C in prewarmed culture cell specific medium. The number of CFU in the inoculum was determined. Cells were infected with 1 mL of bacterial suspension in cell culture specific medium. After 30 min of contact, unbound bacteria were removed by three washes with 1 mL of cell culture medium and the infection was pursued for 6 h. The number of adherent bacteria was determined at 30 min, 3 h and 6 h.

Long-term colonization of bacteria was performed under flow conditions using FaDu, Calu-3 or HDMEC as previously described [17]. Laminar flow chamber experiments were performed on disposable flow chambers composed of six independent flow channels (μ-Slide VI 0.4 purchased from Ibidi, surface area 0.6 cm^2^ per channel) coated with 5 μg of rat tail collagen type I/cm^2^. Cells were seeded in the six channels at a density of 0.3x10^5^/cm^2^ and incubated for 7 days at 37°C in 5% CO_2_ until confluent. A microscopic examination of the cell layers was performed before each flow assay and only channels with a uniformly confluent layer were used. Prior to infection, cell monolayers grown in μ-Slide were stained with cytoplasmic Cell Tracker Orange CMTMR (Life Technologies) according to the manufacturer’s instructions. The GFP-expressing strains were grown during 2 hours with agitation. The OD_600nm_ was then determined and each strain was adjusted to an OD_600nm_ of 0.1 for the piliated strains and 0.3 for the non-piliated strains. 60 μL of this suspension was used to inoculate triplicate channels of a μ-Slide. Bacteria were allowed to adhere to the monolayers for 1.5 hours without flow for the piliated strains and for 2 hours for the non-piliated strains. At 1.5 or 2 hours postinfection, a continuous flow of cell medium containing 3 μg of vancomycin/mL and, when necessary, 1 mM IPTG (isopropyl-β-D-thiogalactopyranoside) was applied for 18 hours at a constant flow rate of 0.04 mL/min for the piliated strains and 0.02 mL/min for the non-piliated strains using a syringe pump (Harvard Apparatus). The flow chamber was placed in an incubator at 37°C with 5% CO_2_ throughout the experiment.

When indicated, DNAse I (Roche) used at a final concentration of 1 μg/mL in the cell medium [20].

The Ibidi μ-Slide flow chambers allow direct observation with inverse microscopy through its transparent plastic bottom. All microscopic observations and image acquisitions were performed on a Leica SP5 confocal microscope. Images were obtained using a x40/1.3 Plan Apo oil objective lens. At the time of confocal acquisition, the cells were examined using red channel to assess the integrity of the monolayer. Three-dimensional biofilm structures reconstructions were generated using the IMARIS software package (Bitplane AG). Biofilm development was quantified with the COMSTAT computer program using biomass and average thickness parameters [39]. The results are expressed as a percentage of the biofilm produced by the WT strain, which is set to 100%. Values represent the means of three independent experiments, with the acquisition of at least six image stacks per each channel.

In some experiments, after 18 hours of incubation, biofilms were aspirated from the flow chamber using a large gauge needle syringe and used for immunofluorescence or immunogold labelling. In this case most of the biofilm was removed for analysis from the surface of the monolayers, leaving a single layer of bacteria which remained adherent to the apical surface of the cells.

### Antibodies

The N-terminal domains of MDAORF4 were detected using purified rabbit polyclonal antibodies raised against peptides H2N-DGFDAAAIGTQVANV-COOH [7].

Type 4 pili was detected using the 20D9 monoclonal antibody that is specific for the SB pilin variant of strain 2C4.3 [40].

MDAORF5 was detected using rabbit polyclonal antibodies against peptides H2N-CINFLKDMGKVGTD-COOH and H2N-CVTEEGKIIRPERVGD-CONH2 [7].

MDAORF10 was detected using rabbit polyclonal antibodies against peptide H2N-FYQFRHGEPHKLINQE-COOH.

### Immunofluorescence

MDAΦ was detected using IF staining as previously described [38]. Briefly, aspirated biofilms or biofilms on Ibidi chamber were fixed on coverslips for 20 min with a solution of PBS-4% paraformaldehyde (PFA). After 2 washes in PBS, samples were incubated with PBS-NH_4_Cl during 5 min. Then samples were washed twice with PBS-0.1%Triton-1%BSA and incubated for 1 hour with the same solution. MDAΦ were stained using the anti-ORF4 N-ter antibody, used at 1/50 dilution and type IV pilin using the 20D9 antibody used at 1/1,000 dilution. While the bacteria were stained with DAPI (4′,6-Diamidine-2′-phenylindole dihydrochloride) solution at 100 ng/mL, the secondary antibodies, used at 1/400 dilution in PBS-0.1%Triton- 1% BSA, were a goat anti-rabbit antibody labelled with Alexa Fluor 488 (Molecular Probes Life tech) and a goat anti-mouse antibody labelled with Alexa Fluor 546 (Molecular Probes Life tech).

### Quantitative PCR

Oligonucleotides were designed using the Primer Express software (PE Applied biosystems) to obtain amplicons of the same size (S2 Table). Real-time PCR was run on an ABI Prism 7700 sequence detection system (Perkin-Elmer Biosystems) using SYBR Green PCR Master Mix (PE Biosystems), according to the manufacturer’s instructions.

Data analyses for a relative quantification of gene DNA were performed by the comparative Ct (threshold cycle) method according to the manufacturer’s instructions (user bulletin 2 for the ABI PRISM sequence detection system) and published data [41]. The parameter Ct is defined as the cycle number at which fluorescence (which is proportional to the quantity of DNA in the tube during the exponential phase of the PCR) passes the fixed threshold. The relative amount of target after normalization to a chromosomal gene *pgm*, is obtained by 2^(Ct^_orf5_—Ct_pgm_^)^.

### Western-blot

Preparation of protein samples, SDS-PAGE separation, transfer to membranes and immunoblotting were performed using standard molecular biology techniques [42]. Detection of immobilized antigens was performed by chemiluminescence using ECL Plus detection reagents (Amersham).

For quantification, we normalized the signal of each western-blot on the number of bacteria used for the protein preparation. All values were then normalized on the corresponding signal of NADP glutamate dehydrogenase.

### Electronic microscopy: Immunogold-labelling

Immunogold labelling of the MDAΦ and the pili were performed as previously described [7]. After aspiration from the Ibidi chambers of the biomass using a syringe with a large gauge needle, biofilms were resuspended in PBS-4% PFA and adsorbed to the grids for 15 min. The grids were then rinsed twice in PBS and placed sequentially onto drops of the following reagents at room temperature: PBS-50 mM NH_4_Cl (5 min), PBS-5% normal goat serum (5 min), and then the anti-ORF4 N-ter antibody diluted 1/50 in PBS-0.2% gelatine for the MDA or the anti-PilE 20D9 monoclonal antibody diluted 1/100 in PBS-0.2% gelatine for the pili (for 60 min). After five washes in PBS-0.2% gelatine, the grid was placed for 60 min on a drop of goat IgG anti-rabbit IgG conjugated to 8-nm-diameter gold particles and donkey IgG anti-mouse IgG conjugated to 12-nm-diameter gold particles diluted 1/60 in PBS-0.2% gelatine. The grids were then subjected to five washes in PBS-0.2% gelatine, fixed in PBS-1% glutaraldehyde (15 min), and washed twice in distilled water. The grids were then treated with phosphotungstic acid, air-dried and viewed. Image acquisition was performed with a JEOL 1011 transmission electron microscope. For the immunogold labelling of the biofilm of the Z5463*gfp*ΔMDAΔ*pilE* mutant, the goat IgG anti-rabbit IgG was conjugated to 18-nm-diameter gold particles.

## Supporting information

S1 Fig Organization of the MDAΦ bacteriophage. The MDAΦ genome possesses 10 Open Reading Frames: *orf10*, *orf1*, *orf2*, *orf9* are responsible for cytoplasmic replication of the phage, *orf3*, *orf4*, *orf5*, *orf6*, *orf7* are implicated in the morphogenesis of the phage and *orf8* is involved in the assembly of the phage [7]. *orf4* encodes the major capsid protein.
(TIF)

S2 Fig **(A) Infection of SCID mice grafted with human skin.** Three mice were infected with the same amount of WT strain or the isogenic prophage deleted derivative. The quantities of bacteria were evaluated in the blood at 1 and 18 h post intravenous inoculation and at 18h in the graft. Error bars indicate the standard errors of the mean (SEM). NS: not significant *p* value (Student t test). **(B) Competitive index between the WT strain and the deleted strain (ΔMDA) in a SCID mice grafted with human skin model.** The competitive indexes were evaluated in the blood 1 and 18 h after infection and in the graft 18 h after infection. The competitive index was calculated by the ratio of [log (UFC_Z5463ΔMDA_)/log (UFC_WT_) in the blood or in the graft] / [log(UFC_Z5463ΔMDA_)/log(UFC_WT_) of the inoculum]. Errors bars indicate the SEM.
(TIF)

S3 Fig Survival of *N*. *meningitidis* wild type (WT) and the MDAΦ deleted (ΔMDA) derivative in normal human serum (NHS). All bacteria were cultured in CDM supplemented with 1 mg/mL of Cohn fraction IV prepared from human serum. The percentage survival was calculated by determining the number of CFU after 30 min of incubation in 60% human serum (NHS). As expected, the noncapsulated mutant is unable to resist to the normal human serum. At least three independent experiments were performed. Errors bars represent the SEM value. hiNHS means heat inactivated human serum.
(TIF)

S4 Fig Short-term adhesion onto epithelial cells of the WT strain and the prophage deleted isogenic variant (Z5463ΔMDA). Inoculum and number of adherent bacteria on FaDu epithelial cells at 30 minutes, 3 h and 6 h were quantified for the WT strain and the prophage deleted strain. Values are the mean of at least three independent experiments. Errors bars represent the standard errors of the mean (SEM) value.
(TIF)

S5 Fig Growth curves of the wild-type strain (WT) and the prophage deleted isogenic variant (Z5463ΔMDA) in the cell medium. (TIF)

S6 Fig Colonization of epithelial cells (Calu-3) by the wild type and prophage deleted strains. Wild-type (Z5463*gfp*) and Z5463*gfp*ΔMDA strains were grown onto Calu-3 epithelial cells for 18 hours under constant flow. The biomass was quantified using the COMSTAT software. At least three independent experiments were performed. The results are normalized as the percentage of the mean of the biomass of the wild-type strain on living cells, which was set to 100%. Error bars indicate the standard errors of the mean (SEM). **p < 0.0001 (Student t test).
(TIF)

S7 Fig Quantification of the biomass covering cells infected with the WT strain and three isogenic mutants (Z5463*gfp*ΔMDA, Z5463*gfp*Δ*orf1* and Z5463*gfp*Δ*orf9*). Wild type and mutants were grown over FaDu epithelial cells for 18 hours under constant flow. At least three independent experiments were performed. The results are normalized as a percentage of the mean biomass of the wild-type strain, which was set to 100%. Error bars indicate the standard errors of the mean (SEM). **p < 0.001 (One-way ANOVA).
(TIF)

S8 Fig Detection of MDAORF10 (A), MDAORF5 (B) and NADP glutamate dehydrogenase (C) by western-blot on whole bacterial proteins harvested from the inoculum (1) and epithelial cells associated bacteria after 8 hours (2) and 22 hours (3). MDAORF10: 8.4 kDa, MDAORF5: 11.2 kDa, NADP glutamate dehydrogenase: 47.4 kDa. The latter allowed the quantification of total proteins present in each well. After normalization on the inoculum and on the signal obtained with the NADP glutamate dehydrogenase, the signal observed with the MDAORF5 antibody was 5 and 7 times that of the inoculum at 8 and 22h, respectively. The signal observed with the anti MDAORF10 antibody was 9 and 15 times that of the inoculum at 8h and 22h, respectively.
(TIF)

S1 Table Strains used in this study. (DOCX)

S2 Table Oligonucleotides used in this study. (DOCX)
