# Supplementary figures and images for: A virulence-associated filamentous bacteriophage of Neisseria meningitidis increases host-cell colonisation
Source: PLoS Pathog. 2017 Jul 13;13(7):e1006495. doi: 10.1371/journal.ppat.1006495 (PMC5526601; doi:10.1371/journal.ppat.1006495)

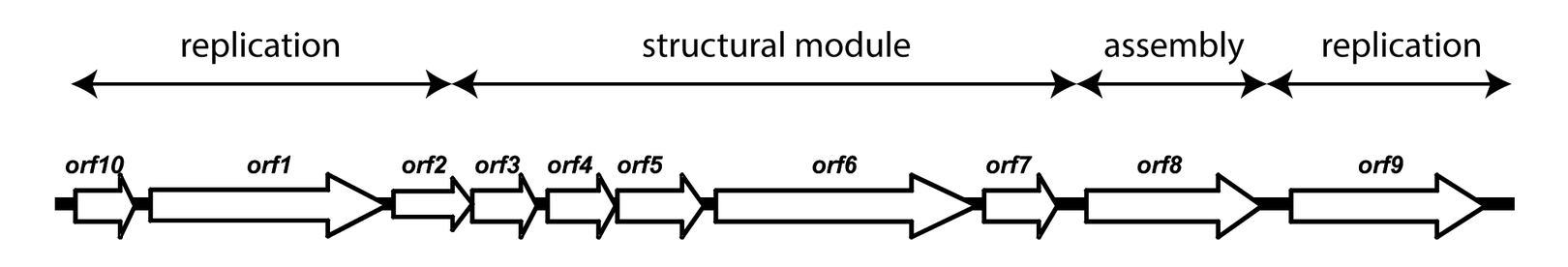

Supplement: S1 Fig — The MDAΦ genome possesses 10 Open Reading Frames: orf10, orf1, orf2, orf9 are responsible for cytoplasmic replication of the phage, orf3, orf4, orf5, orf6, orf7 are implicated in the morphogenesis of the phage and orf8 is involved in the assembly of the phage [7]. orf4 encodes the major capsid protein. (TIF) [file ppat.1006495.s001.tif]

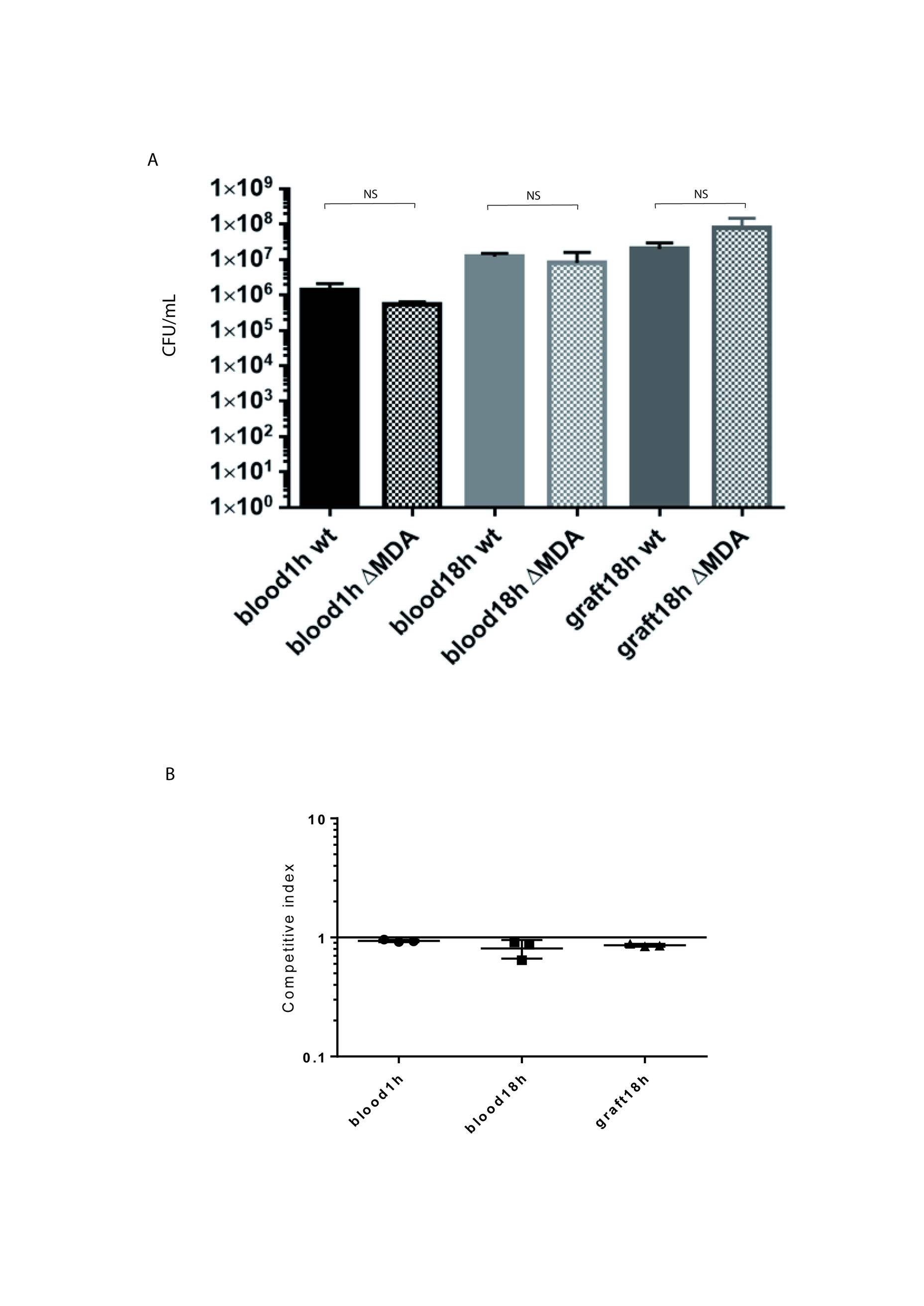

Supplement: S2 Fig — (A) Infection of SCID mice grafted with human skin. Three mice were infected with the same amount of WT strain or the isogenic prophage deleted derivative. The quantities of bacteria were evaluated in the blood at 1 and 18 h post intravenous inoculation and at 18h in the graft. Error bars indicate the standard errors of the mean (SEM). NS: not significant p value (Student t test). (B) Competitive index between the WT strain and the deleted strain (ΔMDA) in a SCID mice grafted with human skin model. The competitive indexes were evaluated in the blood 1 and 18 h after infection and in the graft 18 h after infection. The competitive index was calculated by the ratio of [log (UFCZ5463ΔMDA)/log (UFCWT) in the blood or in the graft] / [log(UFCZ5463ΔMDA)/log(UFCWT) of the inoculum]. Errors bars indicate the SEM. (TIF) [file ppat.1006495.s002.tif]

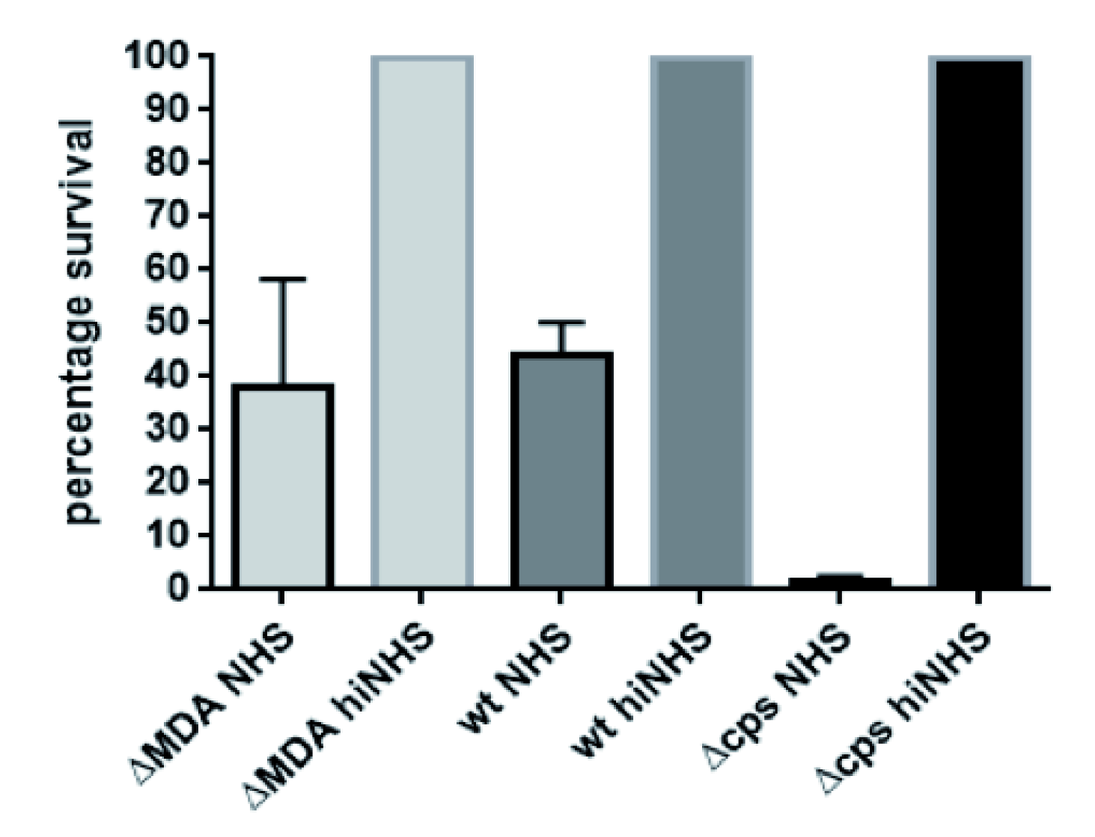

Supplement: S3 Fig — All bacteria were cultured in CDM supplemented with 1 mg/mL of Cohn fraction IV prepared from human serum. The percentage survival was calculated by determining the number of CFU after 30 min of incubation in 60% human serum (NHS). As expected, the noncapsulated mutant is unable to resist to the normal human serum. At least three independent experiments were performed. Errors bars represent the SEM value. hiNHS means heat inactivated human serum. (TIF) [file ppat.1006495.s003.tif]

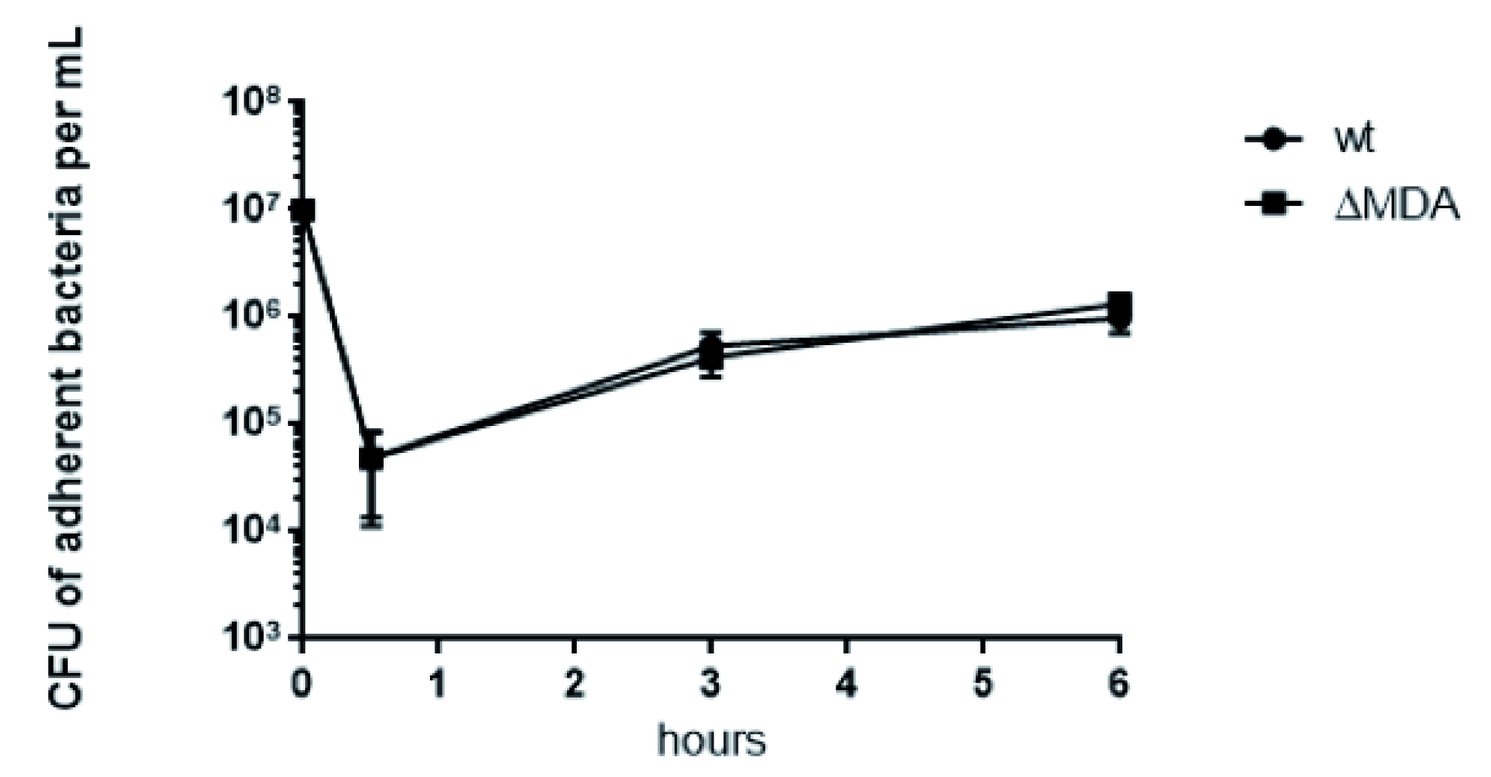

Supplement: S4 Fig — Inoculum and number of adherent bacteria on FaDu epithelial cells at 30 minutes, 3 h and 6 h were quantified for the WT strain and the prophage deleted strain. Values are the mean of at least three independent experiments. Errors bars represent the standard errors of the mean (SEM) value. (TIF) [file ppat.1006495.s004.tif]

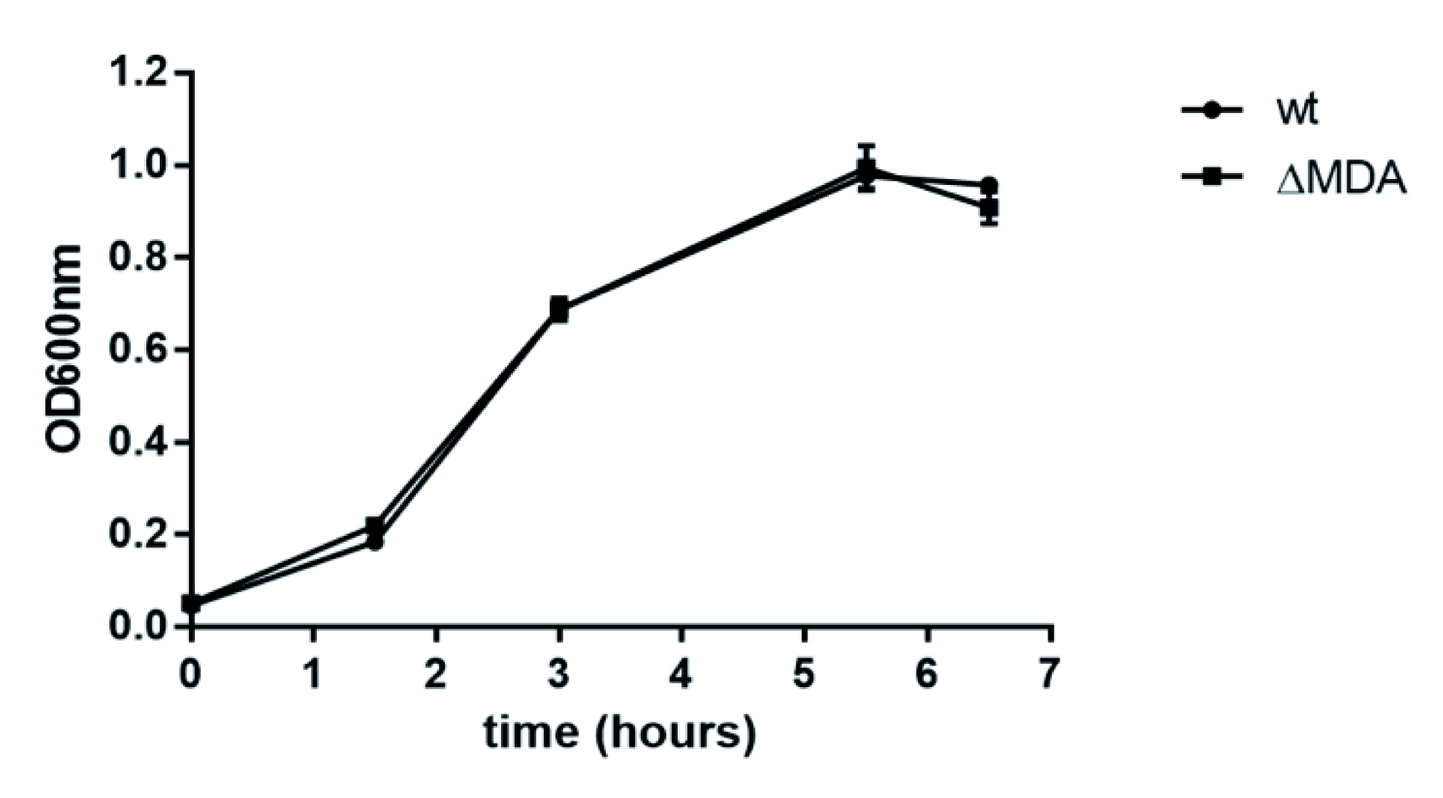

Supplement: S5 Fig — (TIF) [file ppat.1006495.s005.tif]

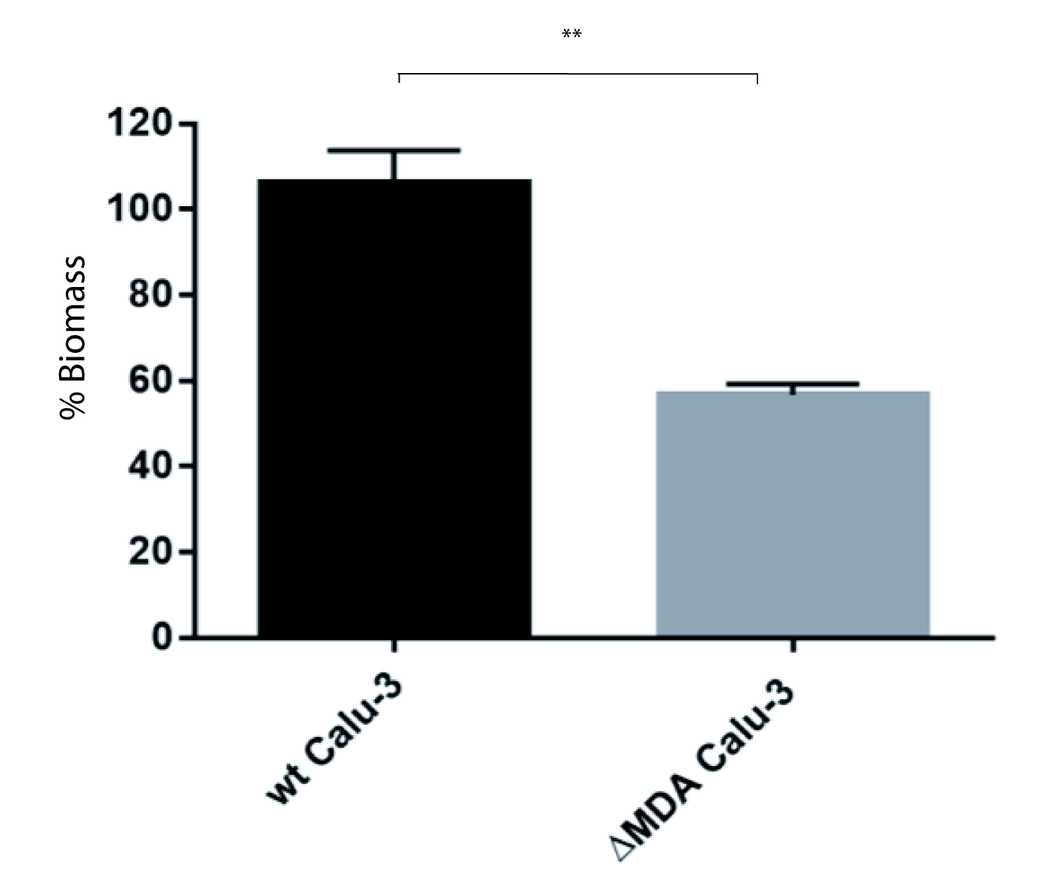

Supplement: S6 Fig — Wild-type (Z5463gfp) and Z5463gfpΔMDA strains were grown onto Calu-3 epithelial cells for 18 hours under constant flow. The biomass was quantified using the COMSTAT software. At least three independent experiments were performed. The results are normalized as the percentage of the mean of the biomass of the wild-type strain on living cells, which was set to 100%. Error bars indicate the standard errors of the mean (SEM). **p < 0.0001 (Student t test). (TIF) [file ppat.1006495.s006.tif]

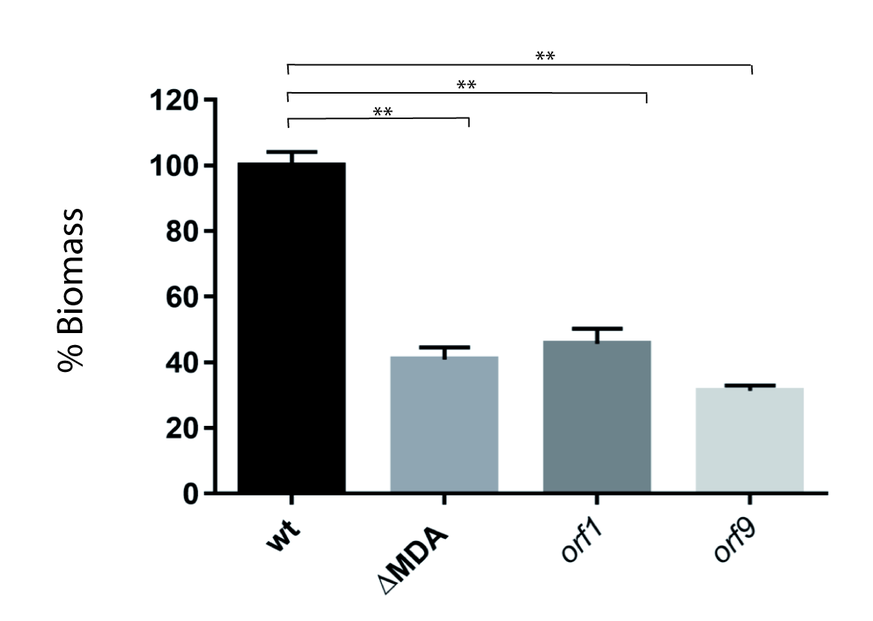

Supplement: S7 Fig — Wild type and mutants were grown over FaDu epithelial cells for 18 hours under constant flow. At least three independent experiments were performed. The results are normalized as a percentage of the mean biomass of the wild-type strain, which was set to 100%. Error bars indicate the standard errors of the mean (SEM). **p < 0.001 (One-way ANOVA). (TIF) [file ppat.1006495.s007.tif]

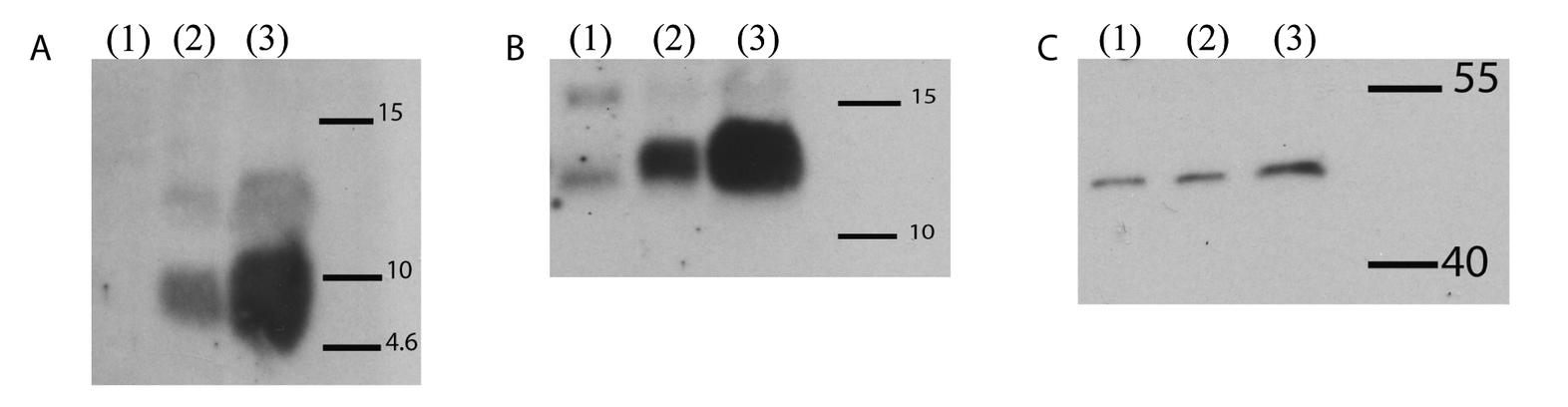

Supplement: S8 Fig — MDAORF10: 8.4 kDa, MDAORF5: 11.2 kDa, NADP glutamate dehydrogenase: 47.4 kDa. The latter allowed the quantification of total proteins present in each well. After normalization on the inoculum and on the signal obtained with the NADP glutamate dehydrogenase, the signal observed with the MDAORF5 antibody was 5 and 7 times that of the inoculum at 8 and 22h, respectively. The signal observed with the anti MDAORF10 antibody was 9 and 15 times that of the inoculum at 8h and 22h, respectively. (TIF) [file ppat.1006495.s008.tif]
