# Supplementary material for: A virulence-associated filamentous bacteriophage of Neisseria meningitidis increases host-cell colonisation
Source: PLoS Pathog. 2017 Jul 13;13(7):e1006495. doi: 10.1371/journal.ppat.1006495 (PMC5526601; doi:10.1371/journal.ppat.1006495)
Supplement: S1 Table — (DOCX) [file ppat.1006495.s009.docx]

**S1 Table. Strains used in this study.**

| **Strains used in this study** | **Description** | **Antibiotic**  **resistance** | **References** |
| --- | --- | --- | --- |
| 2C4.3 | Serogroup C wt strain | / | [33] |
| Z5463 | Serogroup A wt strain | / | [29] |
| Z5463∆MDA | Isogenic deleted strain of the MDA island | Sp | [5] |
| Z5463∆cps | Inactivated *lipA* gene | Km | This study |
| Z5463(SB-*aph*3’) | SB pilin variant expressed in Z5463 | Km | [7] |
| Z5463(SB-*aph*3’)∆MDA | SB pilin variant expressed in isogenic deleted strain of the MDA island | Km, Sp | This study |
| Z5463*gfp* | Z5463 expressing GFP | Cm | This study |
| Z5463*gfp*∆MDA | Isogenic deleted strain of the MDA island expressing GFP | Cm, Sp | This study |
| Z5463*gfp*∆*orf1* | *MDAorf1* mutant | Cm, Km | This study |
| Z5463*gfp*∆*orf9* | *MDAorf9* mutant | Cm, Km | This study |
| Z5463*gfp*∆*pilE* | Z5463 expressing GFP with an inactivated *pilE* | Cm, Em | This study |
| Z5463*gfp*∆MDA∆*pilE* | Isogenic deleted strain of the MDA island expressing GFP with an inactivated *pilE* | Cm, Em, Sp | This study |

Km: Kanamycin, Sp: spectinomycin, Em: erythromycin, Cm: chloramphenicol
