# Supplementary material for: A virulence-associated filamentous bacteriophage of Neisseria meningitidis increases host-cell colonisation
Source: PLoS Pathog. 2017 Jul 13;13(7):e1006495. doi: 10.1371/journal.ppat.1006495 (PMC5526601; doi:10.1371/journal.ppat.1006495)
Supplement: S2 Table — (DOCX) [file ppat.1006495.s010.docx]

**S2 Table. Oligonucleotides used in this study.**

| **Oligonucleotide** | **Sequence (5' to 3')** |
| --- | --- |
| TaqORF5F | ATCGTGTCGGCATGAACTGTT |
| TaqORF5R | CCGGCCTGATGATTTTTCC |
| TaqPGMF | CTATAAAGGCTTGGGCAACG |
| TaqPGMR | GGCAATCAAATCTTGCAGGT |
